# LongDat: an R package for covariate-sensitive longitudinal analysis of high-dimensional data

**DOI:** 10.1093/bioadv/vbad063

**Published:** 2023-05-18

**Authors:** Chia-Yu Chen, Ulrike Löber, Sofia K Forslund

**Affiliations:** Max-Delbrück-Center for Molecular Medicine in the Helmholtz Association (MDC), Berlin 13125, Germany; Experimental and Clinical Research Center, A Cooperation between the Max-Delbrück-Center for Molecular Medicine in the Helmholtz Association and the Charité – Universitätsmedizin Berlin, Berlin 13125, Germany; Charité - Universitätsmedizin Berlin, Corporate Member of Freie Universität Berlin and Humboldt-Universität zu Berlin, Berlin 10117, Germany; DZHK (German Centre for Cardiovascular Research), partner site Berlin, Berlin 10785, Germany; Max-Delbrück-Center for Molecular Medicine in the Helmholtz Association (MDC), Berlin 13125, Germany; Experimental and Clinical Research Center, A Cooperation between the Max-Delbrück-Center for Molecular Medicine in the Helmholtz Association and the Charité – Universitätsmedizin Berlin, Berlin 13125, Germany; Charité - Universitätsmedizin Berlin, Corporate Member of Freie Universität Berlin and Humboldt-Universität zu Berlin, Berlin 10117, Germany; DZHK (German Centre for Cardiovascular Research), partner site Berlin, Berlin 10785, Germany; Max-Delbrück-Center for Molecular Medicine in the Helmholtz Association (MDC), Berlin 13125, Germany; Experimental and Clinical Research Center, A Cooperation between the Max-Delbrück-Center for Molecular Medicine in the Helmholtz Association and the Charité – Universitätsmedizin Berlin, Berlin 13125, Germany; Charité - Universitätsmedizin Berlin, Corporate Member of Freie Universität Berlin and Humboldt-Universität zu Berlin, Berlin 10117, Germany; DZHK (German Centre for Cardiovascular Research), partner site Berlin, Berlin 10785, Germany; Structural and Computational Biology Unit, European Molecular Biology Laboratory, Heidelberg 69117, Germany

## Abstract

**Summary:**

We introduce LongDat, an R package that analyzes longitudinal multivariable (cohort) data while simultaneously accounting for a potentially large number of covariates. The primary use case is to differentiate direct from indirect effects of an intervention (or treatment) and to identify covariates (potential mechanistic intermediates) in longitudinal data. LongDat focuses on analyzing longitudinal microbiome data, but its usage can be expanded to other data types, such as binary, categorical and continuous data. We tested and compared LongDat with other tools (i.e. MaAsLin2, ANCOM, lgpr and ZIBR) on both simulated and real data. We showed that LongDat outperformed these tools in accuracy, runtime and memory cost, especially when there were multiple covariates. The results indicate that the LongDat R package is a computationally efficient and low-memory-cost tool for longitudinal data with multiple covariates and facilitates robust biomarker searches in high-dimensional datasets.

**Availability and implementation:**

The R package LongDat is available on CRAN (https://cran.r-project.org/web/packages/LongDat/) and GitHub (https://github.com/CCY-dev/LongDat).

**Supplementary information:**

Supplementary data are available at *Bioinformatics Advances* online.

## 1 Introduction

Recent years have seen the spawning of high-dimensional data (i.e. data with a large number of features) as biotechnology develops rapidly (Assent, 2012; Witten *et al.*, 2010). For instance, metabolomics, immunomics and metagenomics are becoming prevalent. Among them, count data derived from microbial metagenomics sequencing especially require specific methods to tackle since they have several inherent properties, including uneven sequencing depth across samples, compositional structure, overdispersion and high sparsity (Kodikara *et al.*, 2022; Zhang *et al.*, 2017). When the research aim is to find out the differences in the abundance of microbial features between groups (e.g. time points, treatments), a typical workflow consists of pre-processing and differential abundance analysis steps. Uneven sequencing depth in metagenomic shotgun sequencing needs to be addressed at the pre-processing step because it is known to cause bias in evaluating microbial communities (Sanchez-Cid *et al.*, 2022). Therefore, data-preprocessing approaches such as normalization, transformation and rarefaction have been developed to deal with varying sequencing depth (Lin *et al.*, 2020). When it comes to differential abundance analysis, we need to consider the typical characteristics of microbiome data, including () compositional structure, (ii) overdispersion and (iii) high sparsity. Microbial data from shotgun sequencing are inherently compositional since the total amount of reads each sequencing run can generate is fixed. Thus the abundances of features are not independent of each other (Gloor *et al.*, 2017). Tools have been proposed to account for the compositional structure (Mandal *et al.*, 2015). However, some literature shows that the compositional methods do not always outperform the non-compositional methods (Mallick *et al.*, 2021). Overdispersion denotes a larger variability observed in data than expected from a specific distribution, while high sparsity implies that the data are inflated with zeros. When fitting models to the microbiome data, overdispersion and high sparsity should be addressed. For example, models like negative binomial regression and zero-inflated Poisson regression have been introduced to solve overdispersion and high-sparsity problems (Mallick *et al.*, 2021; Zhang *et al.*, 2017). Collectively, many different microbiome data analysis methods have been proposed so far, and there are plentiful reviews on their comparisons (Calgaro *et al.*, 2020; Swift *et al.*, 2023; Weiss *et al.*, 2017). However, there is no universal method for analyzing all types of microbiome data. Hence, it depends on the characteristics of the data and the user’s research aim to opt for an appropriate method in different scenarios.

Microbiome data can be divided into cross-sectional and longitudinal based on its study design. Cross-sectional data are collected by examining multiple subjects at one time point (Levin, 2006). In contrast, longitudinal data from observational or intervention cohorts are repeated measurements from the same individuals at different time points (Liu *et al.*, 2010). Compared with cross-sectional studies, longitudinal studies account for individual variation and enable researchers to trace changes over time (Hua *et al.*, 2009). Thus, longitudinal data collection has become more common in biological and medical fields nowadays (Liu *et al.*, 2010). When fitting models to longitudinal microbiome data, microbiome features are the dependent variables, while the metadata (i.e. information about the individuals) are the independent variables. The metadata may include the time variable and other variables, such as dietary supplements, changes in meals and weight. The aim of longitudinal microbiome data analysis is to investigate the effect of the time variable since it is the proxy for treatment or intervention in longitudinal data. Accordingly, all variables in the metadata except for the time variables are referred to as covariates, which are defined as the factors other than the variable we are interested in (here the time variable) that might associate with the outcome (here microbiome features) (Field-Fote, 2019). Examples of covariates include, for example, how patients benefiting from a dietary intervention (coded as a time variable) may have their medication dosages reduced during the course of a trial, raising the question of whether observed -omics signature changes are direct effects of the intervention or indirect effects following from the alteration of medication regime (Maifeld *et al.*, 2021). Consequently, to ensure proper analyses of longitudinal microbiome data, it is important to disentangle the time variable's effects from the covariates' effects. In other words, covariates should be uncovered and controlled for to avoid false conclusions (Pedersen *et al.*, 2018). In addition, two other critical points need to be considered when analyzing longitudinal microbiome data. First, selecting appropriate statistical tests from empirical data distributions is important for obtaining proper interpretations (Nahm, 2016). Second, inter-individual variation (e.g. differences in microbial abundance levels between individuals) should be addressed. Several tools dealing with longitudinal microbiome data already exist, and some allow taking in covariates for analysis (Asar *et al.*, 2013; Chen *et al.*, 2016; Gonçalves *et al.*, 2021; Mallick *et al.*, 2021; Mandal *et al.*, 2015; Opgen-Rhein *et al.*, 2021; Timonen *et al.*, 2021). Nevertheless, none of these tools reports explicitly on how the effect of time variable is affected by the presence of other covariates while detecting and simultaneously controlling for them. Therefore, we developed LongDat, an R package capable of performing the tasks as described above.

LongDat is developed and tested centered on microbiome data. However, we extended its utility to work on different data types (e.g. immunome, metabolome, transcriptome). The key to LongDat’s flexibility to adapt to input data with different statistical distributions lies in the utilization of generalized linear models (GLMs) and non-parametric effect size calculations in the pipeline. GLMs can fit skewed data (e.g. data with overdispersion and high sparsity), allow non-constant variances (heteroscedasticity) and model various data types, such as continuous, categorical and ordinal data (Lindsey *et al.*, 1998). To account for inter-individual variation in longitudinal data, we treat sample donor origin (i.e. individual) as a random effect, expanding the GLMs framework to the generalized linear mixed models (GLMMs) (Bolker *et al.*, 2009). LongDat utilizes GLMMs to test the significance of the time variable without and with the presence of covariates in the models, respectively. Subsequently, the effect size of the time variable on each microbiome feature is calculated. In a longitudinal setting, an effect size is defined as the degree of feature difference before and after treatment. Reporting effect sizes in the result is in line with the current best practice, which urges researchers to report effect sizes along with *P*-values in biomedical research (Sullivan *et al.*, 2012). *P*-values from the abovementioned model tests indicate the statistical significance of the time variable (proxy of treatments), whereas standardized and directional effect sizes allow users to interpret the magnitude of effects both manually and within automated frameworks. In the LongDat pipeline, directional non-parametric effect sizes (e.g. Spearman’s rho and Cliff’s delta) are applied to handle both normally and non-normally distributed data (Marfo *et al.*, 2019). LongDat focuses on analyzing monotonic (i.e. the direction of change is fixed) treatment effects within time intervals while reporting covariates for each feature.

In this report, we describe the method of the LongDat R package and validate its performance by comparing it with its closest published counterpart to date, MaAsLin2 (Mallick *et al.*, 2021). We tested the performance of LongDat and MaAsLin2 on simulated, semi-synthetic and real microbiome data. MaAsLin2 is an R package similar to LongDat in several aspects. They both focus on microbiome analysis, adopt GLMMs and allow covariates to be included in the models. The main difference between MaAsLin2 and LongDat is that MaAsLin2 does not explicitly report how the effect of the time variable is affected by the presence of other covariates. In addition, the ways of treating covariates in the two tools differ. MaAsLin2 takes in all covariates along with the time variable into a model at once, while LongDat loops over each covariate in parallel models (see Methods). This distinction leads to significant differences in the results of analyzing longitudinal microbiome data with many covariates, making LongDat more suitable when there are multiple covariates (see Results). Aside from MaAsLin2, we also compared LongDat with other R packages which allow covariates to be included in the analysis, namely ANCOM (Mandal *et al.*, 2015), lgpr (Timonen *et al.*, 2021) and ZIBR (Chen *et al.*, 2016). ANCOM is a microbiome-oriented tool that utilizes the compositional method. Lgpr uses additive Gaussian process regression (a Bayesian method) to achieve non-parametric modeling of longitudinal data. ZIBR incorporates logistic models and zero-inflated Beta regressions with random effects to test the effect of time on microbiome features. Since normalization and rarefaction of the microbial count data might induce considerable changes to the analysis result, we compared the performances of these tools when different normalization or rarefaction techniques were applied, including total-sum scaling (TSS), cumulative-sum scaling (CSS), trimmed mean of M-values (TMM), geometric mean of pairwise ratios (GMPR), centered log-ratio (CLR), rarefaction (Chen *et al.*, 2018; McKnight *et al.*, 2019; Mulè *et al.*, 2022). TSS divides absolute abundance by the total sum of read depth and converts it into relative abundance ranging between 0 and 1. CSS is a quantile normalization method that addresses the bias arising from TSS. The purpose of TMM is to tackle the problem of composition bias and calculate normalization factors that aid in comparing different libraries. GMPR computes the ratio between each pair of values and then takes the geometric mean of those ratios to normalize data. CLR transformation, commonly used for compositional data analysis, takes the logarithm of the ratios of each component to the geometric mean of all components, and then centers the resulting values around zero. Lastly, rarefaction randomly subsamples the data to obtain an equivalent sequencing depth across all samples. Altogether, we assessed how these tools performed with various normalization or rarefaction techniques.

The real microbiome data used in this study for comparison are from our previous study, which reported on a clinical cohort investigating fasting effects on patients with metabolic syndrome (MetS) (Maifeld *et al.*, 2021). By reanalyzing this dataset in which MetS patients benefit from a dietary intervention while medication dosages were altered subsequently during the trial course as an indirect effect following improved health, we demonstrated the need to resolve the time variable (proxy of dietary intervention) and covariate effects in real data. The research question is whether the beneficial outcome is a direct effect of the intervention or an indirect effect following the alteration of the medication regimen. Below we show that LongDat could disentangle the intervention’s beneficial direct effect from the indirect effect of the changes in drug dosage. Finally, to demonstrate LongDat’s flexibility to deal with other datatypes besides microbiome data, we also applied LongDat to the immunome data in the study described above.

## 2 Methods

### 2.1 The LongDat method

**Fig. 1.**
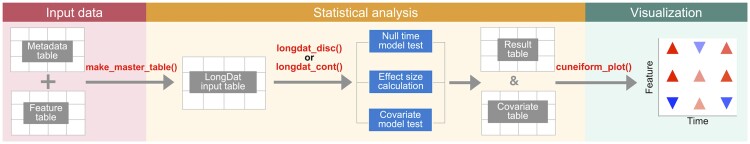
LongDat pipeline overview. Flowchart of LongDat showing the major functions (bold text in red) and steps. The function ‘make_master_table()’ creates a master table that can be taken as input by joining metadata and feature tables provided by the user. The two functions ‘longdat_disc()’ and ‘longdat_cont()’ both perform covariate-sensitive analyses. The main components of them are the null time model test, the effect size calculation and the covariate model test, applied to input master tables to perform covariate-aware tests for the significance of the time variable (the proxy of treatment). The function ‘longdat_disc()’ is suitable for data where time is discrete (e.g. a before/after treatment dataset), whereas ‘longdat_cont()’ is for data with time represented as a continuous variable (e.g. day). Finally, the function ‘cuneiform_plot()’ generates a summarizing plot of the result table. For more detailed tutorials of LongDat application, please visit GitHub (https://github.com/CCY-dev/LongDat), or refer to its vignettes

The LongDat pipeline comprises three major steps, namely the null time model test, covariate model test and effect size calculation (Fig. 1).

1. *Null time model test.* We test whether time associates significantly with each feature (dependent variable), regardless of covariates. LongDat incorporates several R packages specializing in GLMMs, such as MASS, lme4 and glmmTMB (Bates *et al.*, 2015; Brooks *et al.*, 2017; Venables *et al.*, 2002), providing high flexibility for input data types. A negative binomial model is used to fit count data composed of integers (Ver Hoef *et al.*, 2007), such as numbers of sequencing reads. A beta model is applied to proportion data (i.e. that range between 0 and 1) (Ferrari *et al.*, 2004). For binary data (consisting of either 0 or 1), binary logistic regression is performed (Nick *et al.*, 2007). For ordinal data (where the features correspond to ranks), a proportional odds model is adopted (Liu, 2009). Finally, continuous data are first normalized and then fitted by linear models. Each model is a random intercept model with the sample donor origin treated as a random factor to account for between-individual variability and non-independence of samples from the same donor. *P*-values are adjusted for multiple testing using the Benjamini-Hochberg method or other approaches (Benjamini *et al.*, 1995).
2. *Covariate model test.* If covariates are present in a dataset, this step identifies them and disentangles their effects from those of variables of interest (here, the time variable). In the metadata, covariates that exhibit significant association with each feature (e.g. microbiome abundance) via non-parametric tests (i.e. the Wilcoxon rank-sum, Kruskal-Wallis or Spearman’s correlation test) are selected. And then, each selected covariate is included one by one as a fixed effect, together with the time variable, in GLMMs to examine whether or not the time associations can be reduced to the influence of each covariate, reflecting the ‘vibration of effects’ (VoE) concept (Tierney *et al.*, 2021). VoE is the degree to which different combinations of independent variables (e.g. adding covariates) change the outcome and assessed significance of a model. The larger the VoE, the less robust the association is between features and independent variables. That is, a true association should remain significant across all model configurations. Significant time-dependent features are subsequently classified as fulfilling conditions of ‘effect not reducible to covariate’, ‘entangled with covariate’ or ‘effect reducible to covariate’ according to these model tests. If the time variable remains a significant predictor in all models, the feature will be flagged as ‘effect not reducible to covariate’. If the time variable loses significance but the covariate does show significance in any of the models, the feature is marked as ‘effect reducible to covariate’. If there is no clear covariate but at least one model in which the covariate and time both failed to show significance, the feature will be labeled ‘entangled with covariate’.
3. *Effect size calculation.* Non-parametric effect size calculations are implemented. These are Spearman correlation for continuous time variables (e.g. day) and Cliff’s delta for discrete time variables (e.g. before/after treatment) (Macbeth *et al.*, 2010). Effect size calculation is based on a naïve association between the independent variable and the feature, without being partitioned by covariates. Therefore, to ensure LongDat calculates the correct effect size, treatment effects should be monotonic (i.e. no change in the direction of association) within the time interval of the input data. If this is not the case, analyses should be done separately on time subranges of the data for which monotony holds.

All results of the steps mentioned above are summarized into two tables. One table lists the significance estimate (*q*-values adjusted for multiple testing) of time dependence and effect sizes across all features. The other table lists relevant covariates with relative reducibility status for each of them.

### 2.2 LongDat package overview

LongDat is built with R (≥4.0.0), and its dependencies include lme4 (≥1.1-28) (Bates *et al.*, 2015), glmmTMB (≥1.1.3) (Brooks *et al.*, 2017), reshape2 (≥1.4.4) (Wickham, 2007), emmeans (≥1.7.3) (Lenth, 2021), bestNormalize (≥1.8.2) (Peterson, 2021), MASS (≥7.3-56) (Venables *et al.*, 2002), tidyverse (≥1.3.1) (Wickham *et al.*, 2019), effsize (≥0.8.1) (Torchiano, 2020), patchwork (≥1.1.1) (Pedersen, 2020) and car (≥3.0-12) (Fox *et al.*, 2019). There are four main functions that are the most relevant to users of the LongDat package (Fig. 1). For more detailed tutorials for LongDat, please visit GitHub (https://github.com/CCY-dev/LongDat), or install LongDat and then access its vignettes with the command **‘**browseVignettes(“LongDat”)’.

### 2.3 Simulation of longitudinal data with microbiomeDASim

Longitudinal data corresponding to microbiome taxonomic abundance measurements from a cohort were simulated using microbiomeDASim (Williams *et al.*, 2019). MicrobiomeDASim is an R package aimed at simulating longitudinal differential microbiome data. It allows the users to define sparsity, effect size, number of samples and time points. Here, simulated data were generated from a multivariate normal distribution, and the parameter encoding the longitudinal dependency within individuals was a first-order autoregressive correlation structure.

1. *Simulated data with no covariate.* For each sample size (10, 20, 38, 75, 150 and 300) and effect size (Spearman’s rho median ≈ 0.2 or 0.5 for time-varying features) combination, 100 simulations were performed. Each simulated dataset contained 200 features. Among them, 20 features changed over time/under intervention, while the remaining 180 were sampled from the same distribution across time points. Two time points were simulated for each individual.
2. *Simulated data with a single covariate for covariate effect analysis.* A dummy variable correlating with the time variable was manually added to the aforementioned simulated dataset (sample size = 75, effect size median ≈ 0.5). The dummy variable was sampled to correlate with the time variable at a Spearman’s rho of approximately 0.25, 0.5, 0.75 or 0.99. For each correlation level, 100 simulations were performed. For the negative-control data, we shuffled the time variable against all other variables randomly within each individual to wipe out the association between the time variable and the features, and the association between the time variable and the covariates.
3. *Simulated data with multiple covariates.* 1, 2, 4, 8 or 16 dummy variables correlating with the time variable were manually added to the abovementioned simulated dataset (sample size = 75, effect size median ≈ 0.5). The dummy variables were randomly sampled to correlate with the time variable at a Spearman’s rho of approximately 0.25, 0.5, 0.75 or 0.99. For each correlation level, 100 simulations were performed. For the comparison between LongDat and Maaslin2, each dataset contained 200 features (20 changed over time and 180 did not), and for each combination of sample size, effect size and tool, 100 simulations were performed. For the comparison between LongDat, ANCOM, lpgr and ZIBR, each dataset contained 100 features (10 changed over time and 90 did not), and 50 simulations were performed for each combination of sample size, effect size and tool. The reduction of feature number and simulation number was due to the heavy computational resource ANCOM (large memory), lgpr (long runtime) and ZIBR (long runtime) required.

### 2.4 Simulation of longitudinal data with SparseDOSSA2

Besides microbiomeDASim, another set of longitudinal microbial data was simulated using SparseDOSSA2 (Ma *et al.*, 2021). SparseDOSSA2 is an R package specialized for simulating realistic new microbiome data templated on real microbial communities. In these longitudinal data simulations, SparseDOSSA2 adopts generalized linear models to create feature-covariate associations (here, the time variable and other covariates) specified by users, and the template was based on stool microbiome. We followed the tutorial of SparseDOSSA2 (https://github.com/biobakery/biobakery/wiki/SparseDOSSA2) as a complementary approach to the above to simulate longitudinal microbial data for benchmarking.

1. *Simulated data with no covariate.* For each sample size (10, 20, 38, 75, 150 and 300) and effect size (Spearman’s rho median ≈ 0.2 or 0.5 for time-varying features) combination, 100 simulations were performed. Each simulated dataset contained 332 features. Among them, 33 features were spiked (changed over time/under intervention), while the remaining ones were sampled from the same distribution across time points. Two time points were simulated for each individual.
2. *Simulated data with multiple covariates.* 1, 4 or 16 dummy variables correlating with the time variable were manually added to the abovementioned simulated dataset with 332 features and varying sample sizes. The dummy variables were randomly sampled to correlate with the time variable at a Spearman’s rho of approximately 0.25, 0.5, 0.75 or 0.99. For each correlation level, 100 simulations were performed.
3. *Simulated negative control data.* Simulations were done to generate 100 sets of data with two time points, 150 individuals, 332 microbes and zero effect size for all microbial features (i.e. no spiked feature). Six versions of the data with different sequencing depths (the sum of microbial abundance of each feature) were further generated, to test the impact of systematic effects on sampling depth as may occur, for example, in clinical low biomass datasets. Accordingly, for these simulated data, any signal detected will reflect such systematic bias only. In versions one and two of the simulation, both of the total abundances at the first and second time points of each individual were rarefied to 50 000 and 1000, respectively. In version three, the total abundances at the first time point were rarefied to 50 000 and the second time point to 5000, while in version four, the total abundances at the first time point were rarefied to 5000 and the second time point to 50 000. In version five, the total abundances at the first time point were rarefied to 50 000 and the second time point to 1000, while in version six, the total abundances at the first time point were rarefied to 1000 and the second time point to 50 000. When applying LongDat count mode (running negative binomial models), versions three to six were further rarefied to either 5000 or 1000 (as per the lowest sequencing depth) at both time points, such that the sequencing depths are the same between the two time points, reflecting how a user concerned over unequal sampling depths would preprocess the data for this method.

### 2.5 Normalization of the data simulated by SparseDOSSA2

Several R packages were used to normalize or transform the raw simulated microbial features from SparseDOSSA2, TSS, rarefaction (Saary *et al.*, 2017), CLR (van den Boogaart *et al.*, 2008), GMPR (Chen *et al.*, 2018), TMM (Robinson *et al.*, 2010) and CSS (Metwally *et al.*, 2018). The combinations of the tools (LongDat, MaAsLin2, lgpr, ZIBR) and the normalization methods are limited by each tool’s requirement of the input format.

### 2.6 Running LongDat on simulated longitudinal data

The function ‘longdat_cont()’ in the LongDat package was used for analyzing simulated longitudinal data, where the time between samplings was treated as a continuous variable and the data type as count data. The R package peakRAM was adopted to record running time and memory usage (Quinn, 2017). Feature associations with Benjamini-Hochberg (BH)-corrected null-model *q*-value < 0.1 and BH-corrected post-hoc test *q*-values < 0.05 were considered significant. LongDat was run under CentOS Linux 7 and R version 4.1.1 with 8 GB of memory allocated.

### 2.7 Running MaAsLin2 on simulated longitudinal data

The function ‘Maaslin2()’ in the MaAsLin2 package was used for analyzing simulated longitudinal data, where the time between samplings was treated as a continuous variable, and the mode was set as ‘NEGBIN’ for negative binomial model mode and ‘LM’ for linear model mode (Mallick *et al.* 2021). Since MaAsLin2 lacks a covariate model test component that corresponds to the second part of the LongDat pipeline, we ran MaAsLin2 with two separate runs (with and without simulated covariate included as a fixed effect, respectively) for each simulated data with covariates, such that the MaAsLin2 result is comparable with that of LongDat. Features with BH-corrected *q*-value < 0.1 were considered significant. MaAsLin2 was run under CentOS Linux 7 and R version 4.1.1 with 8 GB of memory allocated.

### 2.8 Running ANCOM on simulated longitudinal data

The function ANCOM in the ancom. R Rscript (https://github.com/FrederickHuangLin/ANCOM-Code-Archive) was used for analyzing simulated longitudinal data. The time between samplings was treated as a factor, while the random formula was set as sample ID, and the adjust formula included all the present covariates. Features were considered significant if their W statistics passed the cutoff of the number of taxa multiplied by 0.7. ANCOM was run under CentOS Linux 7 and R version 4.1.1 with 350 GB of memory allocated.

### 2.9 Running ZIBR on simulated longitudinal data

The function ‘zibr()’ in the ZIBR package (Chen *et al.*, 2016) was used for analyzing simulated longitudinal data, where the time between samplings was treated as a continuous variable. All covariates in the data were included for analyses, while subjects and time points were specified. Features with joint *P* values corrected by BH < 0.1 were considered significant. ZIBR was run under CentOS Linux 7 and R version 4.1.1 with 8 GB of memory allocated.

### 2.10 Running lgpr on simulated longitudinal data

The function ‘lgp()’ in the lgpr package (Timonen *et al.*, 2021) was used for analyzing simulated longitudinal data, where the time between samplings was treated as a continuous variable. Sample ID was set as the random effect, and all covariates in the data were included for analyses. The number of drawing samples from a Stan model was 100, and the number of Markov chains was 4. If the time variable was selected using a 95% threshold for the proportion of total explained variance, then the feature was considered significant. Lgpr was run under CentOS Linux 7 and R version 4.1.1 with 8 GB of memory allocated.

### 2.11 Semi-synthetic evaluation of metagenomic data: Fasting study

To perform a semi-synthetic evaluation based on real microbiome data, we selected the first and second time points from the stool microbiome data, using bacterial genus abundances. Next, we randomly shuffled the time variable against all other variables for each individual to eliminate any associations between the time variable and the microbes, as well as between the time variable and the covariates, such that no real signal of the intervention or of the passage of time should remain. Data were normalized or rarefied as the methods in the ‘normalization of the data simulated by SparseDOSSA2’ section.

### 2.12 Evaluation of real metagenomic and immunome data: Fasting study

The fasting study reanalyzed here reported on a clinical cohort investigating fasting effects on patients with MetS (Maifeld *et al.*, 2021). This study has two arms, one being the fasting arm and the other being the DASH (Dietary Approaches to Stop Hypertension, DASH) arm. MetS patients in the fasting arm first underwent seven-day fasting, which consists of two days in which the patients took in a maximum of 1200 kcal/day and five days in which the patients took in 300–350 kcal/day. And then, there was a three-month re-feeding stage where MetS patients were asked to follow the DASH diet. Microbial abundance at the species level and the immunome data of the fasting arm were reanalyzed to demonstrate the value and performance of LongDat.

## 3 Results

### 3.1 LongDat outperforms MaAsLin2 when there are multiple covariates in the simulated data

**Fig. 2.**
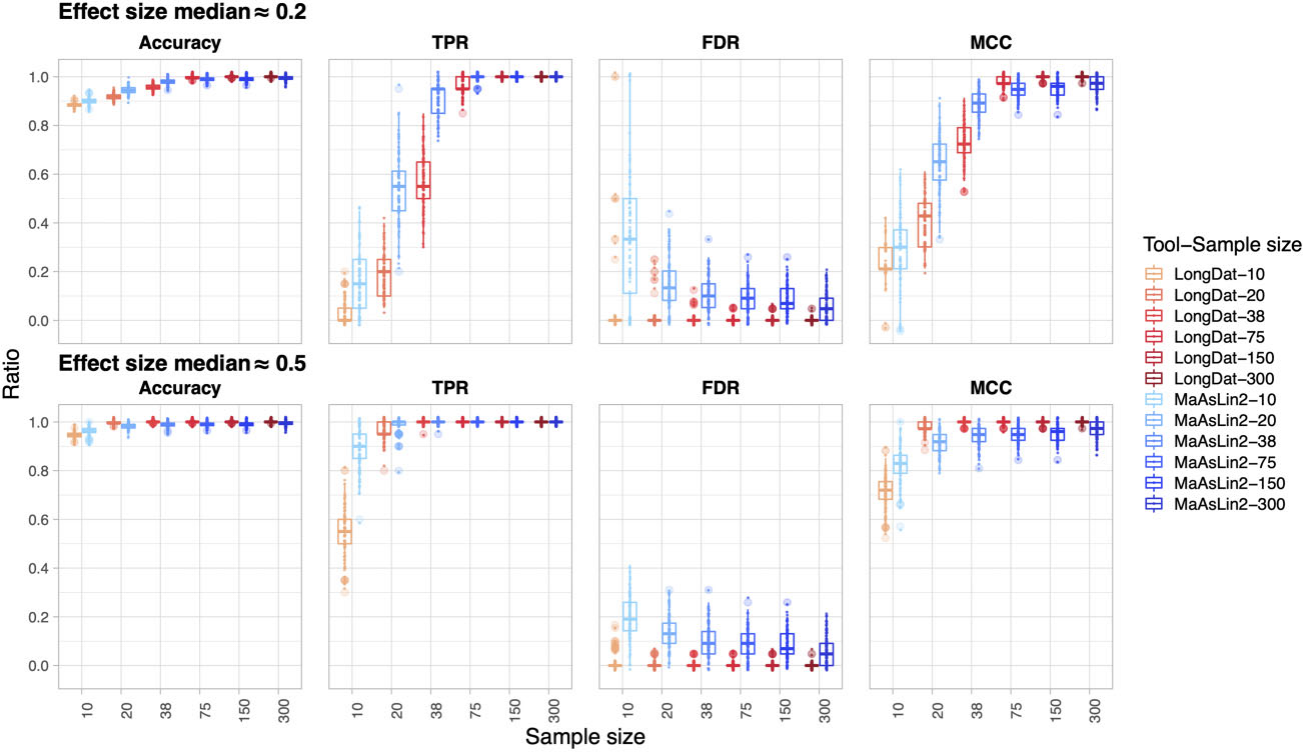
Comparison of LongDat and MaAsLin2 (using the negative binomial model mode) on performance and power applied to microbiomeDASim-simulated longitudinal data. The box plots show the accuracy, true positive rate (TPR), false discovery rate (FDR) and Matthews correlation coefficient (MCC). The upper panel features a low effect size (median ≈ 0.2) while the lower panel features a medium effect size (median ≈ 0.5). For each combination of sample size, effect size and tool, 100 simulations were performed

**Fig. 3.**
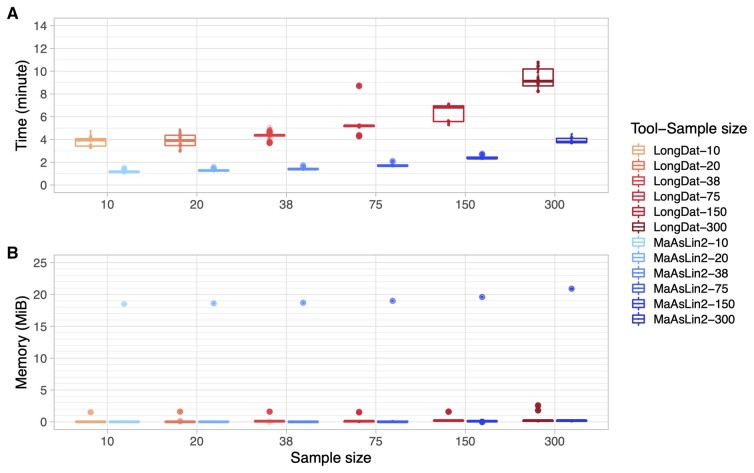
Comparison of LongDat and MaAsLin2 (using the negative binomial model mode) on computational resource profiling applied to microbiomeDASim-simulated longitudinal data. Runtime (**A**) and total used memory (**B**) required by LongDat and MaAsLin2 when run on simulated data with 200 features and various sample sizes. For each sample size, 200 simulations were performed. 1 mebibyte (MiB) ≈ 1.05 megabyte (MB)

First, we compared the performance of LongDat with its closest counterpart up to date, MaAsLin2, by running them on microbiomeDASim-simulated longitudinal datasets templated on microbiome data from cohort studies without covariates. The default setting of MaAsLin2 was doing total-sum scaling (TSS) that converted the data into relative abundance and then fitting linear model (LM). However, this default setting performs worse than using the negative binomial model in terms of accuracy, true positive rate (TPR), false discovery rate (FDR) and Matthews correlation coefficient (MCC) (Supplementary Fig. S1). Therefore, we focused on the MaAsLin2 negative binomial model mode results instead of its default setting. Both LongDat and MaAsLin2 use negative binomial models to fit the data. LongDat and MaAsLin2 have comparable accuracy ranging between 0.9 and 1 (Fig. 2). While MaAsLin2 achieves a higher TPR when sample sizes are small, this comes at the cost of a higher FDR. In contrast, the FDR median of LongDat was controlled at zero across all tested effects and sample sizes, while TPR increases with sample size (Supplementary Fig. S2). LongDat and MaAsLin2 have comparable memory footprints, whereas LongDat has a longer runtime (Fig. 3A and B) due to the additional covariate model test, which MaAsLin2 does not possess. Although MaAsLin2 and LongDat perform similarly on simulated data without covariates, their difference in performance rises as the number of covariates increases in the simulated data (Supplementary Figs S3A, B, S4A and B). The performance of LongDat remains stable across all numbers of covariates. In contrast, FDR escalates and TPR diminishes in MaAsLin2 as the number of covariates increases, especially when more than four covariates are present.

Apart from evaluating LongDat and MaAsLin2 using microbiomeDASim-simulated data, we utilized SparseDOSSA2, another microbiome data simulation tool, to validate that the benchmarking outcome of LongDat is consistent between different simulation tools. Here, we assessed the performance of LongDat and MaAsLin2 using various modes, such as linear or negative binomial models, on simulated data that was either raw, normalized or rarefied. The benchmarking of performance and requirement of computational resources on SparseDOSSA2-simulated data with 0 (Supplementary Fig. S5A and B), 1 (Supplementary Fig. S6A and B), 4 (Fig. S7 and S7B) and 16 covariates (Supplementary Fig. S8A and B) are presented. Within a broader perspective, these results reproduce our findings in the benchmarking using microbiomeDASim-simulated data, where MaAsLin2 and LongDat perform similarly on simulated data in the absence of covariates. However, as the number of covariates increases, the performance gap between the two methods becomes more evident. The performance of LongDat remains stable across all numbers of covariates while FDR surges and TPR diminishes in MaAsLin2, particularly when more than four covariates are present. Upon closer examination of each normalization method, MaAsLin2's negative binomial model mode generally produces higher TPR but also higher FDR than its linear model mode (except when run on CLR-transformed data, which has a high FDR), thus similar accuracy and MCC. On the other hand, LongDat produces comparable outcomes when either linear or negative binomial models are run on data processed through different normalization or rarefaction methods (except for CLR paired with linear model, which performs the worst). Specifically, we believe rarefaction is necessary for some situations, although the pair of negative binomial model and rarefied count data has higher FDR than the pair of linear model TSS-normalized data. For example, we simulated longitudinal negative control data with two time points in which the sequencing depths varied systematically between the two points. In this scenario, the negative binomial model applied to rarefied count data maintains a near-zero false positive rate (FPR), while the linear model applied to non-rarefied TSS-normalized data results in a high FPR when the depth variation between the two time points was large (Supplementary Fig. S9). Therefore, we conclude that the use of linear models to analyze TSS-normalized data is reasonable only when there is no systematic bias in sequencing depth between groups or time points; otherwise, the analysis may result in many false positives. There is no method suitable for all scenarios; instead, the selection of an appropriate approach highly depends on the data's nature.

### 3.2 LongDat outperforms ANCOM in computational efficiency and outperforms lgpr and ZIBR in overall performance when tested on simulated data

In addition to MaAsLin2, we also compared LongDat with several other tools capable of analyzing longitudinal microbiome data, including ANCOM, lgpr and ZIBR, by examining their performances when multiple covariates are present (Supplementary Figs S10A, B, S11A and B). Here the simulated data were generated by microbiomeDASim. The results show that the FDR of ANCOM remains around zero as LongDat does and has comparable accuracy, TPR and MCC when there are less than four covariates, whereas TPR dwindles as the number of covariates increases. The major drawback of ANCOM is that it needs large memory (requires 350 GB to be allocated or else errors would occur and halt) to run through each simulated dataset (here each containing 100 features), making it challenging to run ANCOM on a large scale. ZIBR does not perform well because its FDR is high, and its accuracy, TPR and MCC are low across all numbers of covariates. Moreover, ZIBR needs much time to run through each dataset with large sample sizes (e.g. around 55 h to finish a run with 300 samples and 16 covariates). Lastly, while lgpr has better TPR than LongDat when the sample size is small (≤38), it simultaneously has a high level of FDR. The primary shortcoming of lgpr is that its run time (e.g. around 65 h with 150 samples and 1 covariate) is substantially longer than all other tools, and it could not finish any run within the time limit (96 h) of the high-performance computing cluster we used. This impedes lgpr from being widely applied to microbiome data in a common research environment. From these results, we concluded that LongDat is the ideal tool for data with multiple covariates since it has decent performance and low computational resource requirements (Supplementary Table S1).

As we did with the comparison between LongDat and MaAsLin2, we employed SparseDOSSA2 to assess the performance of LongDat, lgpr and ZIBR, in addition to the microbiomeDASim-simulated data comparison. ANCOM's high memory requirement (>350 GB) for SparseDOSSA2-simulated data analysis caused it to fail to run on the high-performance computing cluster we utilized, leading to its exclusion from the analysis here. The assessment of computational resource requirements and performance benchmarking using SparseDOSSA2-simulated data with varying numbers of covariates (0, 1, 4 and 16) is depicted in Supplementary Figures S12A, B, S13A, B, S14A, B, S15A and B, respectively. These findings confirm our earlier observations based on microbiomeDASim-simulated data. ZIBR, which operates on TSS-normalized data, has a high FDR and takes a long time to execute. lgpr performs better than LongDat in terms of TPR for small sample sizes, regardless of normalization or rarefaction, but it also has a high FDR. The primary disadvantage of lgpr is that it takes significantly longer to run than any of the other tools, making it not able to complete any run within the time limit (96 h) of the high-performance computing cluster we used when the sample size is relatively large. The performances of both ZIBR and lgpr decline as the number of covariates increases. In contrast, LongDat maintains decent performance and computational resource requirements regardless of the number of covariates.

### 3.3 The performance of LongDat was evaluated using semi-synthetic data, demonstrating a low false positive rate

To estimate the performance of LongDat based on data aside from simulated data (microbiomeDASim and SparseDOSSA2), we conducted a semi-synthetic evaluation as well. In this evaluation, we shuffled the time variable against all other variables in the fasting microbiome data (at the genus level). Next, we compared the ratio of significant associations between unshuffled and shuffled data. The result of LongDat indicates few to no false positives in the shuffled data in all modes, regardless of whether covariates were included or excluded in the analysis (Supplementary Fig. S16). Conversely, MaAsLin2, lgpr and ZIBR were all influenced by the presence of covariates in their analyses. MaAsLin2 identifies fewer significant associations in the unshuffled data and more false positives in the shuffled data than LongDat. ZIBR and lgpr result in much higher false positive rates than LongDat. Consistent with prior analyses, we confirmed that LongDat is robust against false positive findings in both simulated and semi-synthetic evaluations.

### 3.4 LongDat provides a more smooth and more efficient workflow than MaAsLin2 despite similar performance

**Fig. 4.**
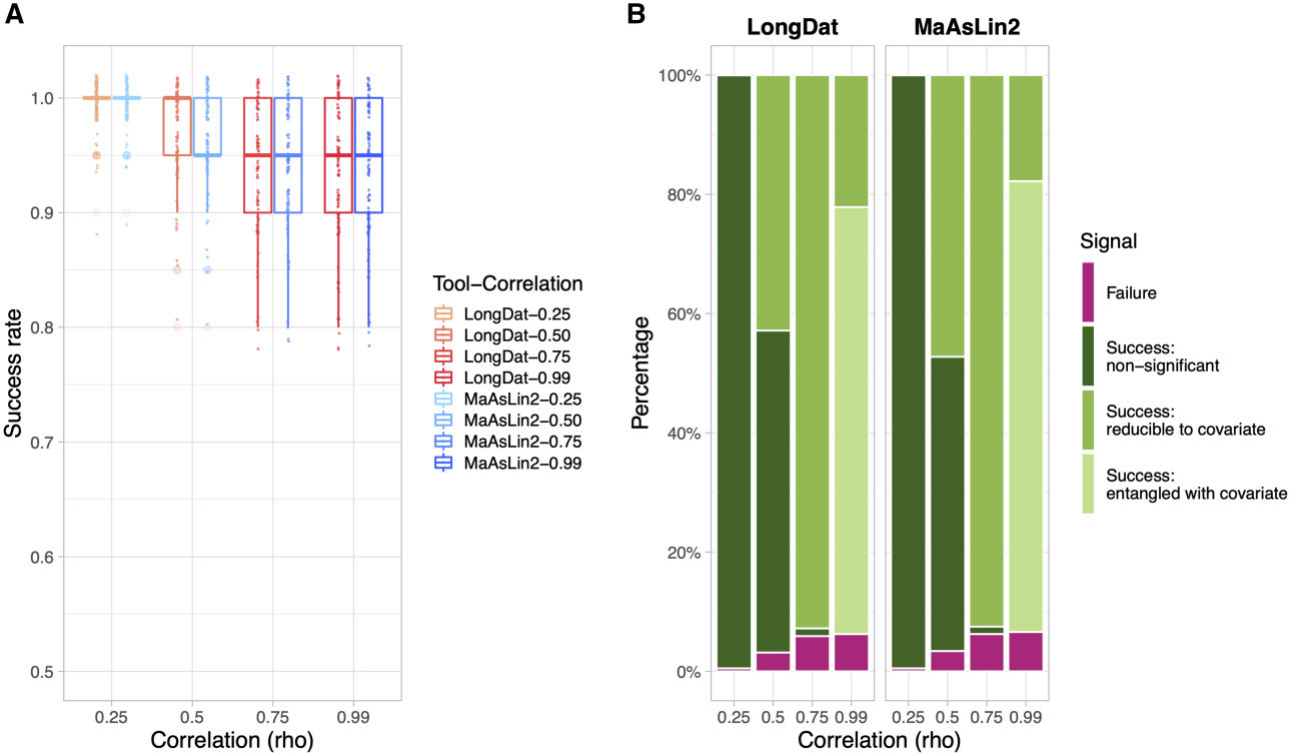
Comparison of LongDat and MaAsLin2 on covariate-sensitive analysis applied to microbiomeDASim-simulated longitudinal data. To compare the ability of LongDat and MaAsLin2 on detecting covariates, a dummy variable was added to the simulated data and was tested for its effect. ‘Success’ is defined as a tool indicating the dummy variable as either ‘non-significant’, ‘reducible to covariate’ or ‘entangled with covariate’ (time is the covariate here), whereas ‘failure’ occurs when a tool wrongly concludes that the dummy variable is significant and the time variable is not. (**A**) The box plot shows the success rate of using LongDat and MaAsLin2 to filter out and conclude covariate effects at different degrees of correlation between the dummy variable and time. (**B**) The bar chart illustrates the proportion of success and failure across correlation degrees. For each degree of correlation, 100 simulations were done

Since MaAsLin2 is the only alternate tool where memory and runtime scaling do not preclude its application to our full benchmark, the analyses below focus on the comparison between MaAsLin2 and LongDat. To compare the performance of LongDat and MaAsLin2 on detecting covariate effects, we added a dummy variable correlating with the time variable and tested whether this dummy variable, when instead used as the time input variable, was wrongly reported as exhibiting a relationship to the simulated feature independent of time (Fig. 4A and B). LongDat requires only a single run for this test. In contrast, MaAsLin2 lacks a covariate model test component corresponding to the LongDat pipeline's second part, so we ran MaAsLin2 in two separate runs (with and without covariate included in the model, respectively) to generate comparable results with LongDat. We found that LongDat and MaAsLin2 achieved similar performance, with the median success rate for both tools in correctly concluding covariate effects remaining at ∼0.95 (as per FDR threshold/family-wise error rate) even when the dummy and time variables were strongly correlated. As a negative control, we tested how LongDat performs when both the features and covariates are not associated with time by shuffling the time variable randomly within each individual (Supplementary Fig. S17). Here we tested if the shuffled time variable was reported as significant. We found no false positive occurred across all correlation degrees (the correlation between the time variable and covariate was, in fact, destroyed because time was shuffled). These results demonstrate that both LongDat and MaAsLin2 perform well in accuracy and correctly flagging covariates.

In the simulated data, with rho being as high as 0.99 between the dummy and time variables, we demonstrated that LongDat could distinguish data generated from different underlying ground truths even when there is substantial confounding. It is worth noting that LongDat does not label the dummy variable as ‘non-significant’ (i.e. having no relationship with the feature at all) but rather as ‘reducible to covariate’ or ‘entangled with covariate’ when the correlation degree is high. These two labels do not signify that the dummy variable is irrelevant to the features but instead intend to communicate that its association with the features might be from an indirect association. In real data, however, it will be more challenging to discern between the dummy and the time variables if they are highly correlated. In that case, more investigation or experiments are required to confirm which variable has direct versus indirect influence, necessitating more careful inspection and domain knowledge before robust conclusions can be drawn.

### 3.5 LongDat performs more robustly than MaAsLin2 in real data with multiple covariates

**Fig. 5.**
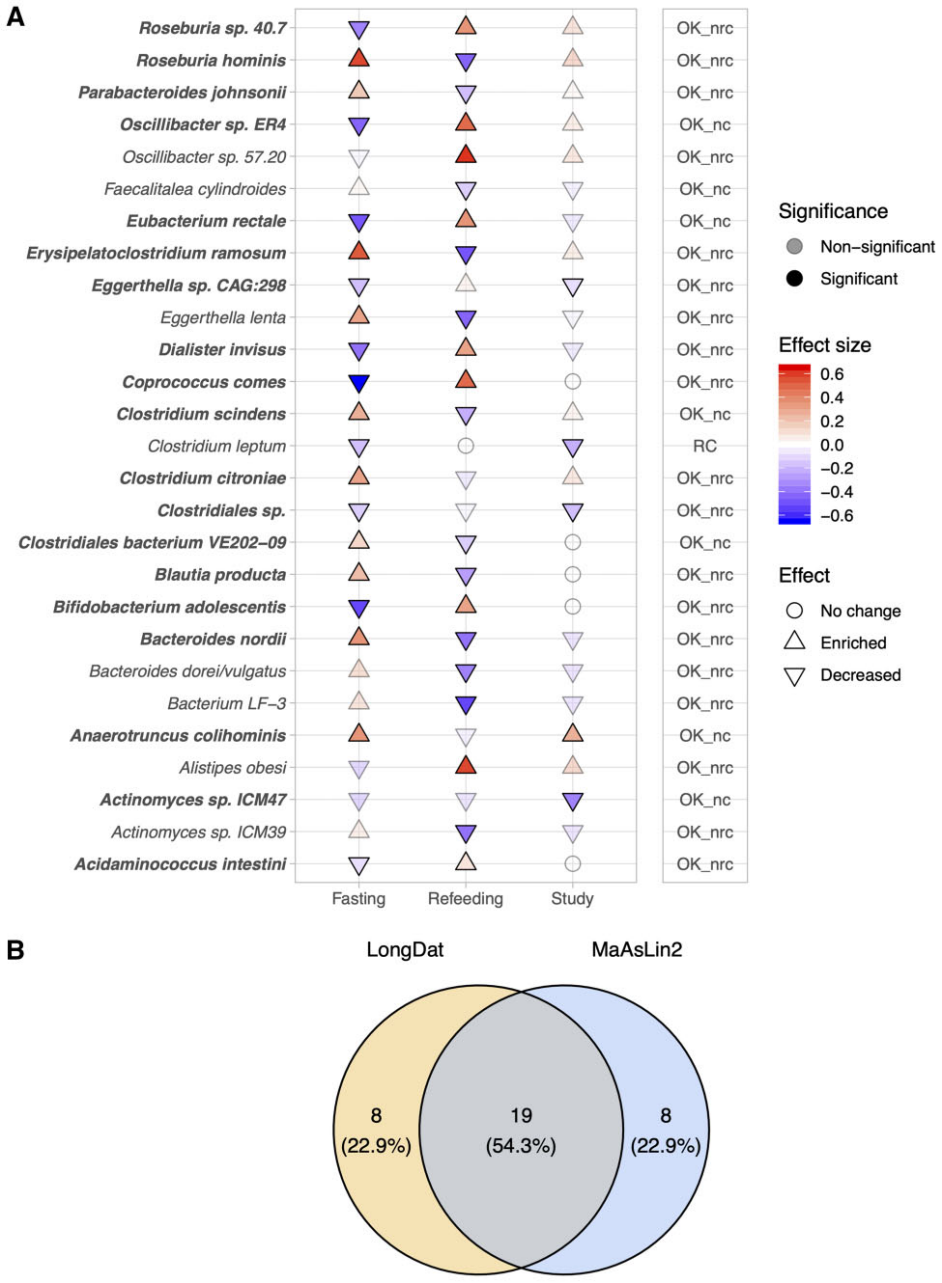
Comparison of LongDat and MaAsLin2 on analyzing real microbiome data. (**A**) The cuneiform plot on the left panel shows the gut microbes (species level) that display significant differences in their abundance in at least one of the time intervals in the assessed study (Maifeld *et al.*, 2021). In that study, metabolic disease patients underwent 7-day fasting and then a 3-month re-feeding period. In the plot, ‘fasting’ indicates the time elapsed from Day 0 to 7, while ‘refeeding’ indicates Day 7 to 90, and ‘study’ indicates the overall duration from Day 0 to 90. Bold species are the ones that appeared significant in both LongDat and MaAsLin2 results. The right panel reports the covariate status of each microbe as follows. *OK_nc*: OK and no covariate. Time/intervention has significant effect and there is no covariate. *OK_nrc*: OK and not reducible to any covariate. Time/intervention has significant effect, and its effect is independent of all tracked covariates. *RC*: Effect reducible to covariate. Time/intervention when tested on its own achieves significance; however, its effect is better explained by that of a covariate (whereas the inverse is not true). Microbes with BH-corrected model test q-values < 0.1 and BH-corrected post-hoc test q-values < 0.05 are regarded as significant. (**B**) The Venn diagram shows the significant species found by LongDat and MaAsLin2. Numbers indicate counts and percentages of each category. Note that for MaAsLin2, covariates were not included in the analyses here. When covariates were included in MaAsLin2 analysis, only 2 of the species showed significance here remained significant, while all others returned error due to NA in *P*-values and standard errors (Supplementary Table S5)

Subsequently, to compare the performance of LongDat and MaAsLin2 on real data, we reanalyzed gut microbial data from a clinical cohort investigating fasting effects on patients with MetS (Maifeld *et al.*, 2021). One aim of the study was to investigate how fasting affects blood pressure while controlling for changes in medication. When no covariates (i.e. medication variables) were included in the MaAsLin2 analysis, LongDat and MaAsLin2 each identified 27 microbial species that were significantly altered in abundance throughout the intervention, with 19 of them being consistent between the two (Fig. 5A and B; Supplementary Tables S2, S3 and S4). However, when covariates were included, only two species were reported significantly different in abundance by MaAsLin2 (Supplementary Table S5). This is because models fitting most of the significantly affected species returned errors (showed NA in *P*-values and standard errors) when multiple fixed effects were included. LongDat avoids this problem by having only one covariate in a model at one time and loops over all of them. Thus, LongDat performs more robustly than MaAsLin2 for this type of data when multiple covariates are present in the dataset, but at the cost of higher runtime.

### 3.6 LongDat can be applied to other data types, such as immunome data

Finally, to demonstrate that LongDat can run on other data types besides microbiome data, we applied LongDat to the immunome data from the same fasting study mentioned above (Supplementary Figs S18 and S19; Supplementary Tables S6–S9). The immunome data consist of proportion (percentage) and non-proportion (non-percentage) data, so they were analyzed by LongDat proportion mode and measurement mode, respectively. These results show that LongDat can tackle other types of data in addition to microbiome counts.

## 4 Discussion

We introduce LongDat, an R package that analyzes longitudinal data for intervention (or treatment) effects while accounting for covariates throughout the intervention. LongDat was developed and tested as a microbiome analysis tool, and we made it able to work on other high-dimensional data types by using flexible and robust approaches, chiefly GLMMs and non-parametric tests. The resulting output allows convenient downstream analysis and interpretation. Instead of proposing a new theory or mathematical tool to deal with the problems of high-dimensional data, multiple covariates and different data types (distribution), we packaged the GLMMs and non-parametric tests together to automate the process of analyzing high-dimensional data. LongDat relieves the users of the burden of having to program the R linear modeling functions from scratch. With the standardized output, including effect sizes reported in Cliff’s delta, and the visualization of the significances and influences of covariates for each feature, it is easier for biologists to integrate, compare and visualize their findings. Though here we do not present a theoretical advance, we believe LongDat is a practical and convenient modeling tool immediately applicable to the scale of problems faced, e.g. in cohort- or intervention-centered systems medical research, and therefore promotes high-dimensional data analysis in the biomedical research field. One limitation of LongDat is that it reports standardized non-parametric effect sizes of discrete (Cliff’s delta) or continuous (Spearman’s rho) variables, both of which are calculated independently of other covariates. Thus, when covariates are present in the data, the reported effect size is not a perfect estimate as it does not incorporate covariates for calculation. We currently are not aware of any partial standardized directional effect size metrics that would let us circumvent this obstacle, but we are actively searching for them to include in later versions of LongDat.

By conducting simulations using two independent tools, namely microbiomeDASim and SparseDOSSA2, we confirm that the statements below are consistent across benchmarking platforms. The advantages of LongDat over the most similar existing tool, MaAsLin2, include lower FDR, the ability to explicitly report covariate effects and their effect sizes, and thus a more convenient functionality in reporting and describing covariates for each feature. The latter, which reports the influence of covariates on the tested variable, is the exclusive feature of LongDat that no other tool possesses to the best of our knowledge. The other important feature of LongDat is that it can handle multiple covariates at once and maintain stable performance. This feature is achieved by having only one covariate in a model at one time and looping over them instead of including all covariates at once, which forms a lengthy formula. This approach grants LongDat robustness when facing multiple covariates, but at the cost of higher runtime. By contrast, the performance of MaAsLin2 is highly dependent on the number of covariates in the data. While MaAsLin2 and LongDat have comparable accuracy when there are no covariates, we observe a trade-off between TPR and FDR in them. Hence, MaAsLin2 and LongDat are suitable in different scenarios depending on the priorities of the application. For instance, when a lower FDR is more emphasized in an analysis, or when there are many covariates in a study, LongDat will be a better choice. In contrast, if a higher TPR and power are prioritized, and no covariate is in the data, then MaAsLin2 is a good option.

In addition to MaAsLin2, we also compared LongDat with three other tools: ANCOM, lgpr and ZIBR. While the performance of ANCOM is fairly good when the number of covariates is low, ANCOM suffers from the need for huge memory to run. On the other hand, the performances of both lgpr and ZIBR are highly dependent on the number of covariates, and both tools suffer from long runtime. Furthermore, ANCOM and lgpr rely on arbitrary cutoffs (W statistics and the proportion of total explained variance, respectively), which might pose inconvenience and confusion to the users. Despite the unfavorable ZIBR results shown here, we believe in the potential of the zero-inflated models (represented by ZIBR here) since they characterize the distribution of highly sparse microbiome data well. We will keep track of its development (or other related zero-inflated models) and plan to integrate it into the future version of LongDat.

To investigate the impact of normalization and rarefaction on analysis results, we conducted benchmarking tests on each tool using different preprocessing methods. Interestingly, we find that LongDat and MaAsLin2 have similar overall performance (based on accuracy and MCC) when analyzing data without any covariates, irrespective of the modes (negative binomial or linear model) and preprocessing methods (except for the CLR transformation). However, the discrepancy in the performance of LongDat and MaAsLin2 widens as the number of covariates increases. While all modes of MaAsLin2 struggle to maintain high true positive rates and low false discovery rates with four or more covariates, LongDat remains stable and performs well across all scenarios. Meanwhile, ZIBR and lgpr did not excel in this round of benchmarking test because of high FDR and extremely long runtime that impedes the users from applying them effectively. Furthermore, by simulating datasets with high variation in sequencing depth between time points (or groups), we underscored that the choice of normalization or rarefaction technique should be tailored to the specific characteristics of the data. In the example above, rarefaction is required to remove the systematic bias between time points, which leads to false positive findings. Ultimately, we believe that there is no one-size-fits-all approach to data preprocessing and analysis, and researchers should carefully select appropriate protocols according to the unique features of their data.

In conclusion, our comprehensive benchmarking, including independent simulation tools, semi-synthetic and real data evaluations, demonstrates that the LongDat package we present here is a computationally efficient and low-memory-cost analysis tool for longitudinal data with multiple covariates, and facilitates robust biomarker searches in high-dimensional datasets.

## Supplementary Material

vbad063_Supplementary_Data

## Data Availability

The scripts for simulating longitudinal data, running LongDat, ANCOM, lgpr, ZIBR and MaAsLin2, and also the raw data for the fasting study are available at https://github.com/CCY-dev/LongDat_paper_supplement.git. The stool sequencing data of the fasting study are deposited on NCBI under the accession PRJNA698459.
