# Supplementary material for: LongDat: an R package for covariate-sensitive longitudinal analysis of high-dimensional data
Source: Bioinform Adv. 2023 May 18;3(1):vbad063. doi: 10.1093/bioadv/vbad063 (PMC10284677; doi:10.1093/bioadv/vbad063)
Supplement: vbad063_Supplementary_Data [file vbad063_supplementary_data.zip › Supplements.pdf]

Supplementary material

LongDat: an R package for covariate-sensitive longitudinal analysis of high-dimensional data

Chia-Yu Chen[1, 2, 3, 4], Ulrike Löber[1, 2, 3, 4], Sofia K. Forslund[1, 2, 3, 4,5,*]
[1]Experimental and Clinical Research Center, a joint cooperation of Max Delbrück Center for Molecular Medicine and Charité - Universitätsmedizin Berlin, 13125 Berlin, Germany. [2]Charité - Universitätsmedizin Berlin, corporate member of Freie Universität Berlin and Humboldt-Universität zu Berlin, 10117 Berlin, Germany. [3]DZHK (German Centre for Cardiovascular Research), partner site Berlin, 10785 Berlin, Germany. [4]Max Delbrück Center for Molecular Medicine in the Helmholtz Association (MDC), 13125 Berlin, Germany. [5]Structural and Computational Biology Unit, European Molecular Biology Laboratory, 69117 Heidelberg, Germany

Supplementary Tables

Supplementary table 1. Summary of comparison of all tools

	TPR	FDR	TPR and FDR affected by the number of covariates	Memory requirement	Speed	Able to include covariates in analysis	Provides covariate effect of each feature	Multiple test correction	Needs* arbitrary cutoff
LongDat	Low when sample size is small	Generally low	Not affected	Small	Medium	Yes	Yes	Yes	No
MaAsLin2 (Mallick et al. 2021)	Low when there are multiple covariates	High when there are multiple covariates	Highly affected	Small	Fast	Yes	No	Yes	No
ANCOM (Mandal et al. 2015)	Low when sample size is small	Generally low	Affected	Large	Medium	Yes	No	Yes	Yes**
ZIBR (Chen et al. 2016)	Low when there are multiple covariates	Generally high	Highly affected	Small	Slow	Yes	No	Yes	No
lgpr (Timonen et al. 2021)	Low when sample size is small	High when sample size is small	Highly affected	Medium	Slow	Yes	No	No	Yes***

*Indicates if the user needs to select an arbitrary threshold to decide the significance of the tested variable on a feature.
**Relies on W statistics, which is the number of each feature is tested to be significantly different against other features.
***Relies on the proportion of total explained variance in a model.

Supplementary table 2. The result table of LongDat applied to the fasting study microbial species data.

Supplementary table 3. The covariate table of LongDat applied to the fasting study microbial species data.

Supplementary table 4. The result table of MaAsLin2 applied to the fasting study microbial species data. No covariates (medication variables) were included in the run.

Supplementary table 5. The result table of MaAsLin2 applied to the fasting study microbial species data. Covariates (medication variables) were included in the run.

Supplementary table 6. The result table of LongDat applied to the fasting study immunome proportion data.

Supplementary table 7. The covariate table of LongDat applied to the fasting study immunome proportion data.

Supplementary table 8. The result table of LongDat applied to the fasting study immunome non-proportion data.

Supplementary table 9. The covariate table of LongDat applied to the fasting study immunome non-proportion data.

Supplementary Figure

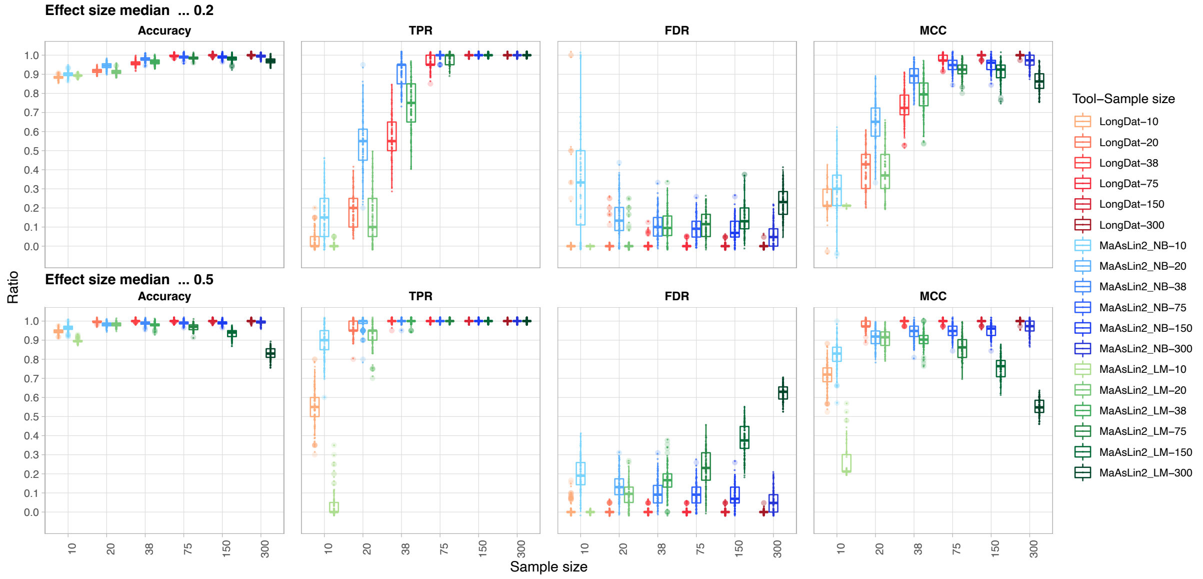

Fig. S1. Comparison of LongDat (using the negative binomial model) and MaAsLin2 (using the negative binomial model or total-sum scaling (TSS)/linear model modes) on performance and power applied to microbiomeDASim-simulated longitudinal data. The box plots show the accuracy, true positive rate (TPR), false discovery rate (FDR), and Matthews correlation coefficient (MCC). The upper panel features a low effect size (median ≈ 0.2) while the lower panel features a medium effect size (median ≈ 0.5). NB: negative binomial model; LM: TSS and linear model. For each combination of sample size, effect size, and tool, 100 simulations were performed.

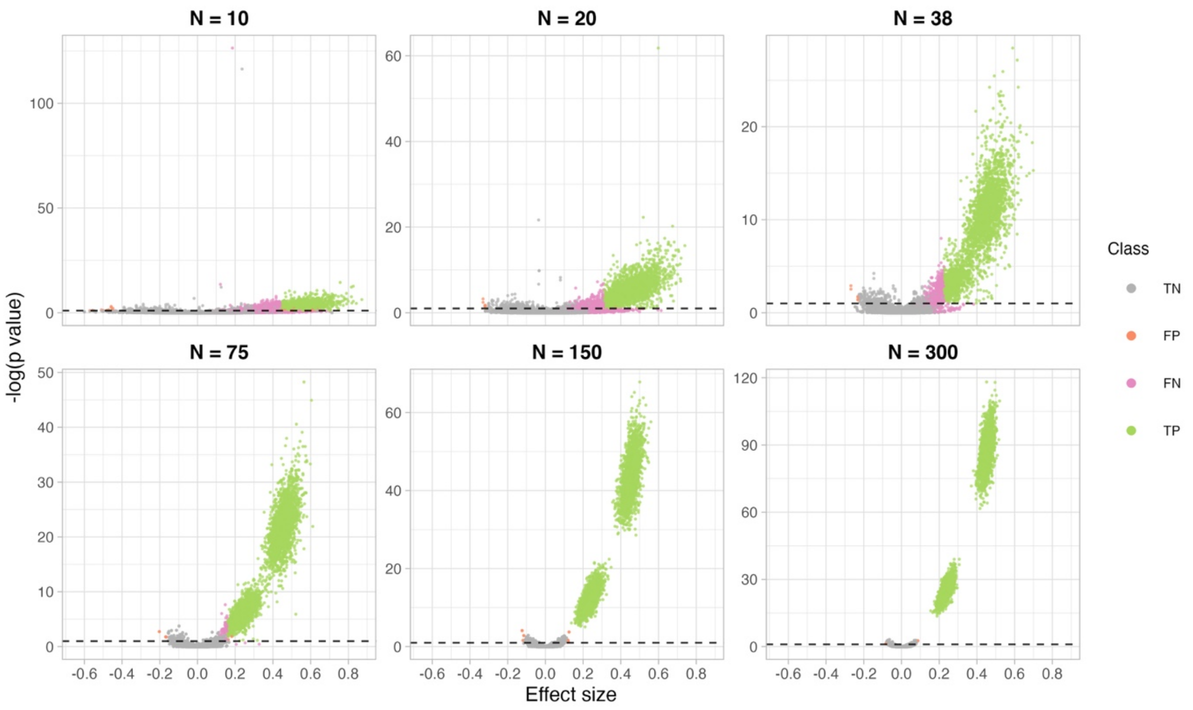

Fig. S2. Volcano plot showing type I and type II errors in LongDat analysis results on microbiomeDASim-simulated data. The vertical axis shows -log value of p-values from the model test corrected by the Benjamini-Hochberg procedure (BH), and the horizontal axis shows the effect size (Cliff's delta). The dashed lines indicate the significance level (BH-corrected $p < 0.1$) for the model test. The decision on concluding significance is based on both the model test and the following post-hoc test. The significance threshold is set at 0.1 for the BH-corrected model test p-value and 0.05 for the BH-corrected post-hoc test p-value. For each sample size, 200 simulations were performed. N represents the sample size. TN: true negative; FP: false positive; FN: false negative; TP: true positive.

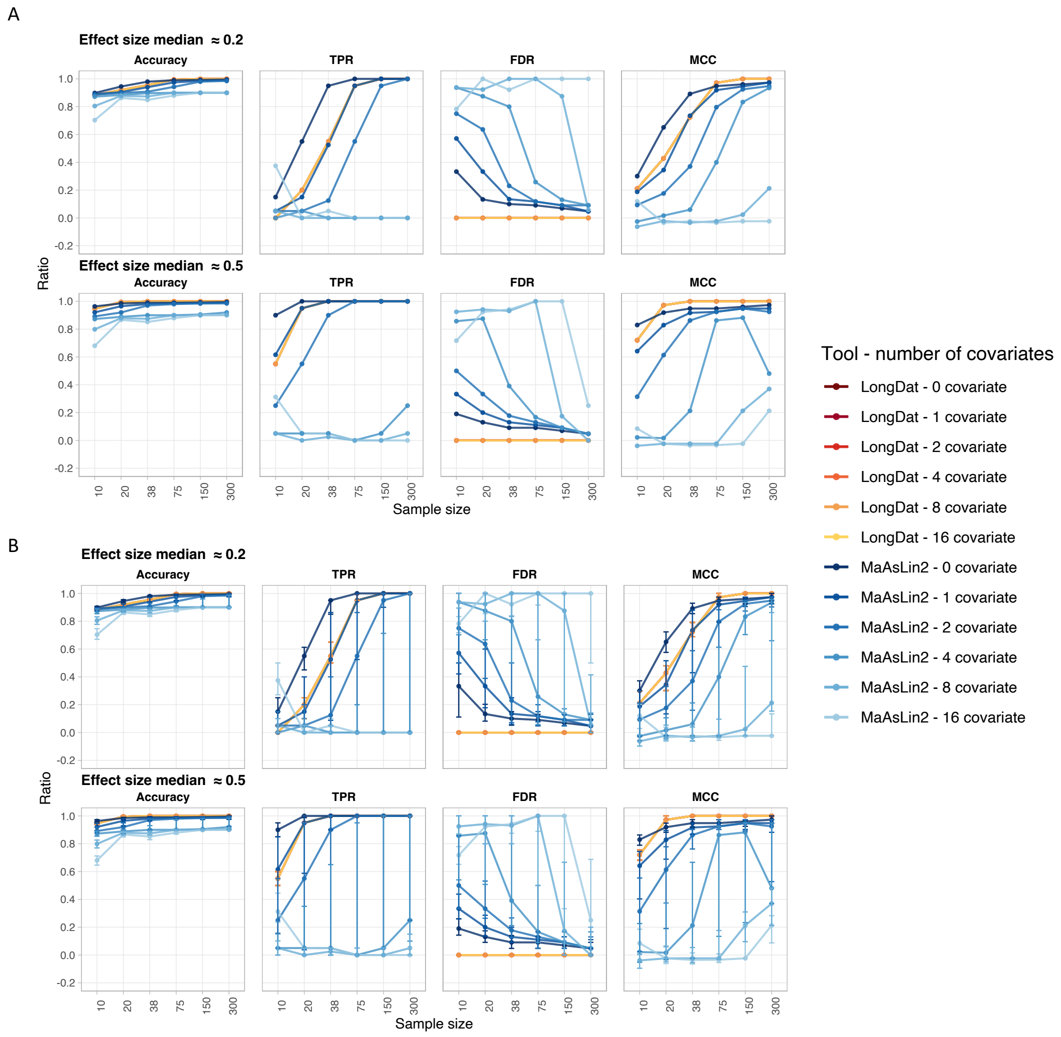

Fig. S3. Comparison of LongDat and MaAsLin2 (negative binomial model) on performance and power applied to microbiomeDASim-simulated longitudinal data with multiple covariates. The line plots show the medians of accuracy, true positive rate (TPR), false discovery rate (FDR), and Matthews correlation coefficient (MCC) without (A) and with (B) the first and third quartiles. The lower bars are the first quartiles, while the upper bars are the third quartiles. For each combination of sample size, effect size, covariate number, and tool, 100 simulations were performed.

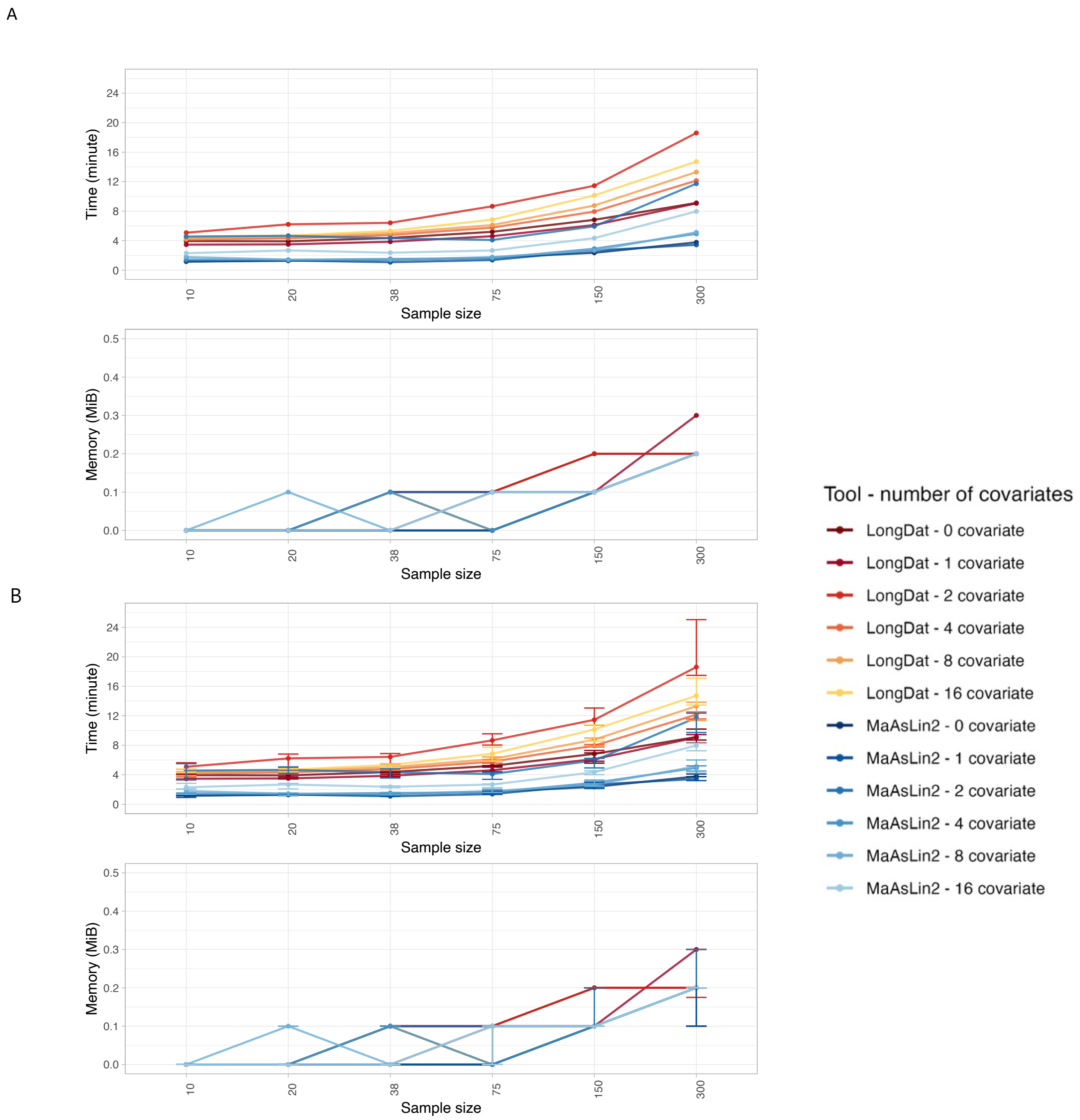

Fig. S4. Comparison of LongDat and MaAsLin2 (negative binomial model mode) on computational resource profiling applied to microbiomeDASim-simulated longitudinal data with multiple covariates. Line plots show the medians of runtime and total used memory required by LongDat and MaAsLin2 when run on simulated data with 200 features and various sample sizes without (A) and with (B) the first and third quartiles. The lower bars are the first quartiles, while the upper bars are the third quartiles. For each sample size, 200 simulations were performed. 1 mebibyte (MiB) ≈ 1.05 megabyte (MB).

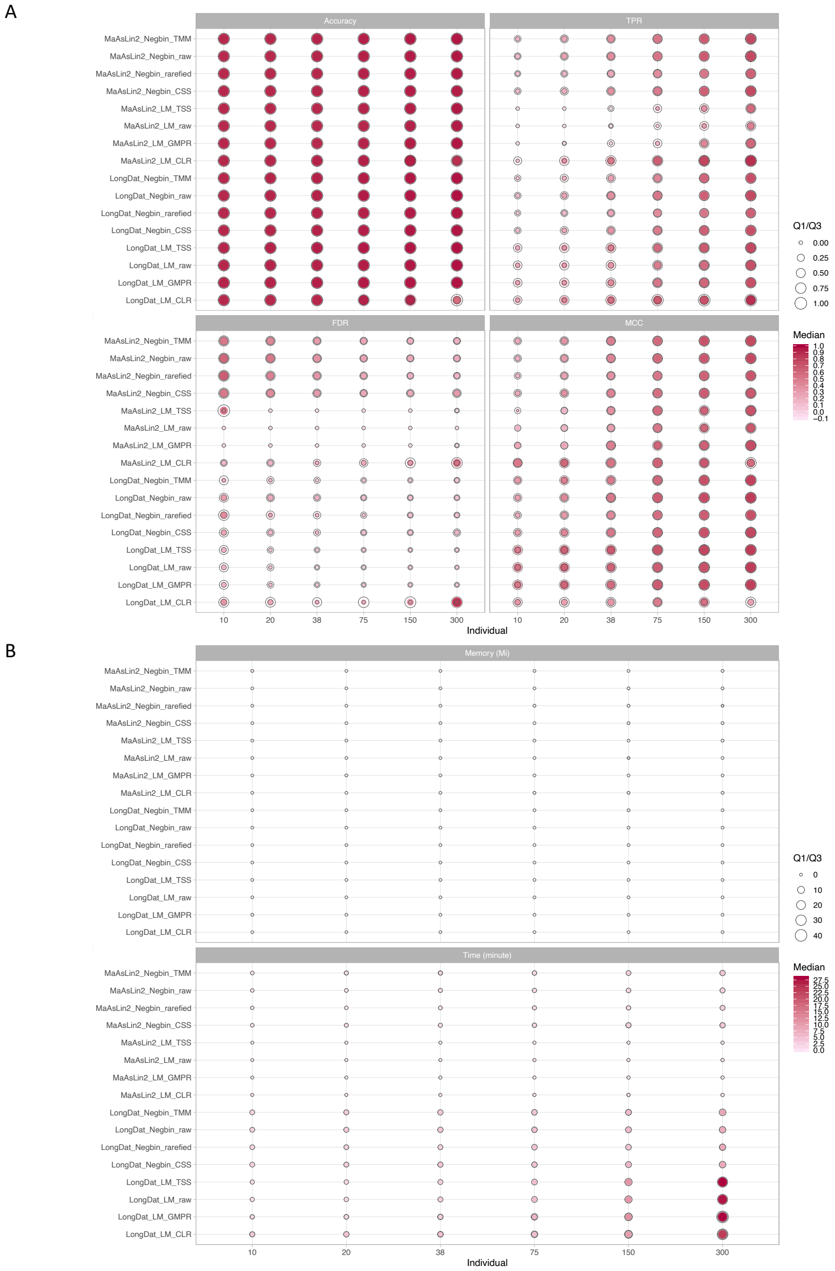

Fig. S5. Comparison of different modes of LongDat and MaAsLin2 on performance and computational resource profiling applied to SparseDOSSA2-simulated longitudinal data with no covariate. The circles show the first quartiles, medians and third quartiles of (A) accuracy, true positive rate (TPR), false discovery rate (FDR), and Matthews correlation coefficient (MCC), and (B) runtime and total used memory. The filled circles (red) are the medians, while the inner and outer hollow circles are the first and third quartiles, respectively. For each sample size, 200 simulations were performed (100 simulations of effect size median ≈ 0.2 Spearman's rho and 100 simulations of effect size median ≈ 0.5 Spearman's rho for time-varying features). 1 mebibyte (MiB) ≈ 1.05 megabyte (MB). Negbin: negative binomial model; LM: linear model; TMM: trimmed mean of M-values; raw: raw counts; rarefied: rarefied counts; CSS: cumulative-sum scaling; TSS: total-sum scaling; GMPR: geometric mean of pairwise ratios; CLR: centered log-ratio.

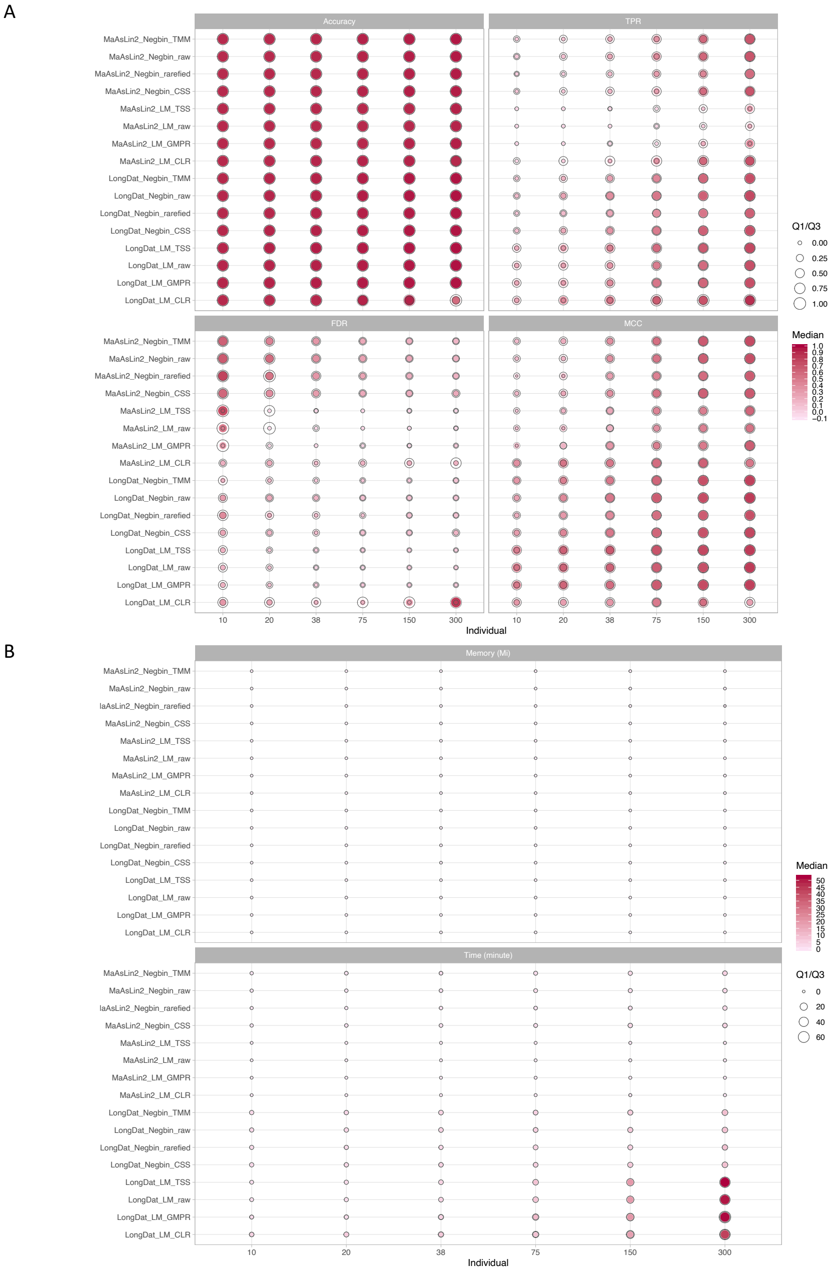

Fig. S6. Comparison of different modes of LongDat and MaAsLin2 on performance and computational resource profiling applied to SparseDOSSA2-simulated longitudinal data with 1 covariate. The circles show the first quartiles, medians and third quartiles of (A) accuracy, true positive rate (TPR), false discovery rate (FDR), and Matthews correlation coefficient (MCC), and (B) runtime and total used memory. The filled circles (red) are the medians, while the inner and outer hollow circles are the first and third quartiles, respectively. For each sample size, 200 simulations were performed (100 simulations of effect size median ≈ 0.2 Spearman's rho and 100 simulations of effect size median ≈ 0.5 Spearman's rho for time-varying features). 1 mebibyte (MiB) ≈ 1.05 megabyte (MB). Negbin: negative binomial model; LM: linear model; TMM: trimmed mean of M-values; raw: raw counts; rarefied: rarefied counts; CSS: cumulative-sum scaling; TSS: total-sum scaling; GMPR: geometric mean of pairwise ratios; CLR: centered log-ratio.

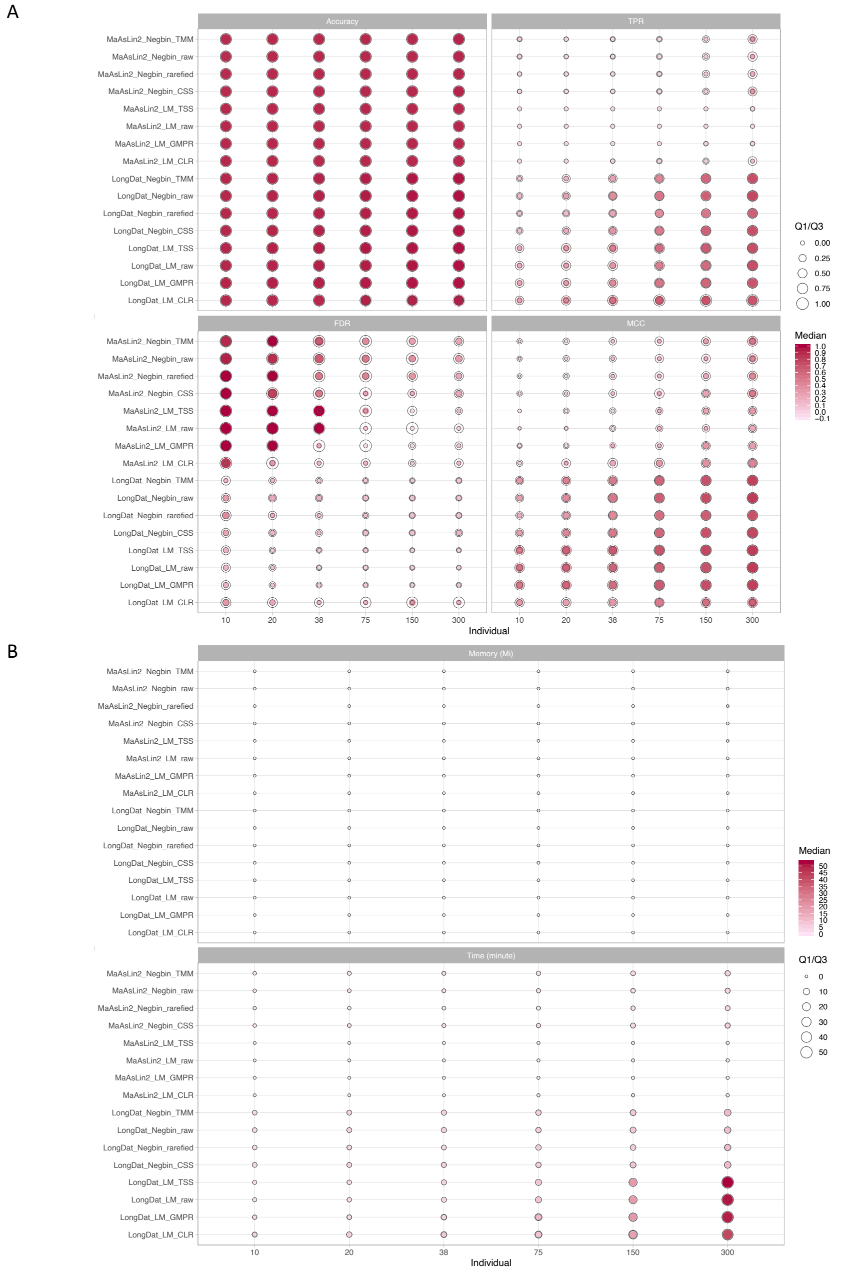

Fig. S7. Comparison of different modes of LongDat and MaAsLin2 on performance and computational resource profiling applied to SparseDOSSA2-simulated longitudinal data with 4 covariates. The circles show the first quartiles, medians and third quartiles of (A) accuracy, true positive rate (TPR), false discovery rate (FDR), and Matthews correlation coefficient (MCC), and (B) runtime and total used memory. The filled circles (red) are the medians, while the inner and outer hollow circles are the first and third quartiles, respectively. For each sample size, 200 simulations were performed (100 simulations of effect size median ≈ 0.2 Spearman's rho and 100 simulations of effect size median ≈ 0.5 Spearman's rho for time-varying features). 1 mebibyte (MiB) ≈ 1.05 megabyte (MB). Negbin: negative binomial model; LM: linear model; TMM: trimmed mean of M-values; raw: raw counts; rarefied: rarefied counts; CSS: cumulative-sum scaling; TSS: total-sum scaling; GMPR: geometric mean of pairwise ratios; CLR: centered log-ratio.

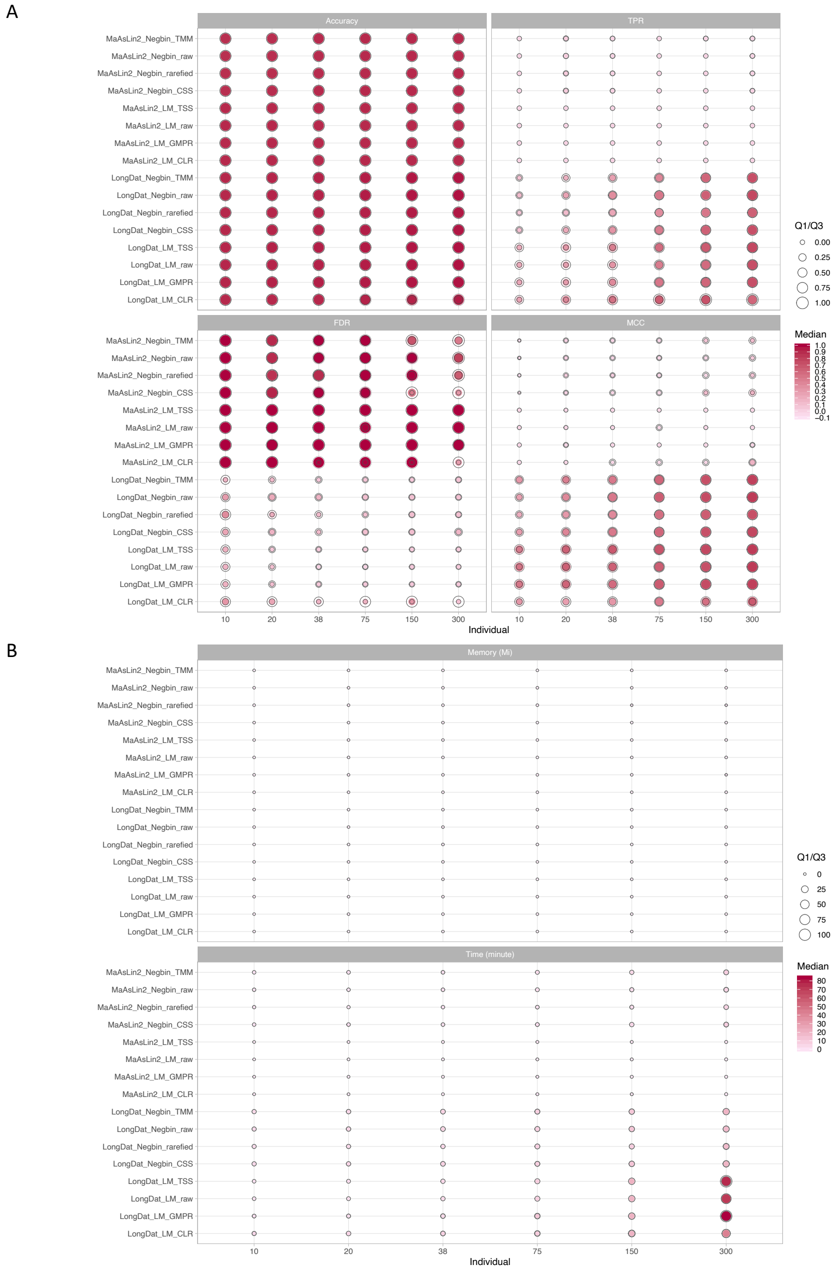

Fig. S8. Comparison of different modes of LongDat and MaAsLin2 on performance and computational resource profiling applied to SparseDOSSA2-simulated longitudinal data with 16 covariates. The circles show the first quartiles, medians and third quartiles of (A) accuracy, true positive rate (TPR), false discovery rate (FDR), and Matthews correlation coefficient (MCC), and (B) runtime and total used memory. The filled circles (red) are the medians, while the inner and outer hollow circles are the first and third quartiles, respectively. For each sample size, 200 simulations were performed (100 simulations of effect size median ≈ 0.2 Spearman's rho and 100 simulations of effect size median ≈ 0.5 Spearman's rho for time-varying features). 1 mebibyte (MiB) ≈ 1.05 megabyte (MB). Negbin: negative binomial model; LM: linear model; TMM: trimmed mean of M-values; raw: raw counts; rarefied: rarefied counts; CSS: cumulative-sum scaling; TSS: total-sum scaling; GMPR: geometric mean of pairwise ratios; CLR: centered log-ratio.

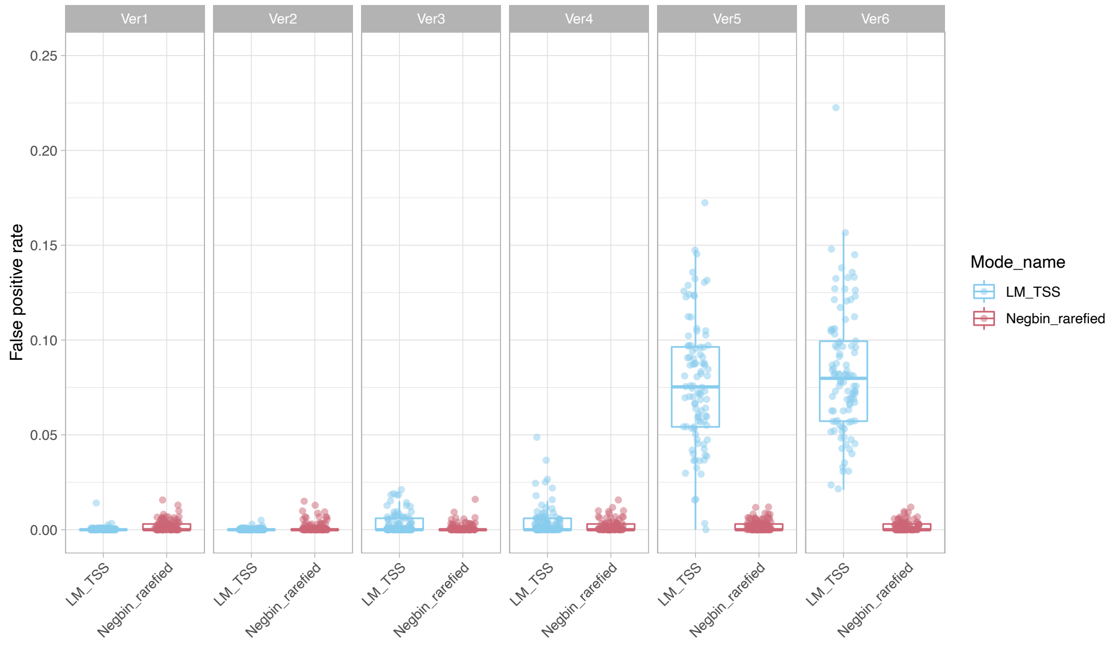

Fig. S9. Comparison of the false positive rate (FPR) of different modes of LongDat applied to SparseDOSSA2-simulated negative control longitudinal data with two time points, 150 individuals, 332 microbes (effect size is zero for all features, i.e., no time-varying feature). Linear model paired with TSS normalization, and negative binomial model paired with rarefied count are compared. For each pair of tool and mode, 100 simulations were performed. Version one to six were generated based on the same raw simulated data. In versions one and two, both of the total abundances at the first and second time points of each individual were rarefied to 50000 and 1000, respectively. In version three, the total abundances at the first time point were rarefied to 50000 and the second time point to 5000, while in version four, the total abundances at the first time point were rarefied to 5000 and the second time point to 50000. In version five, the total abundances at the first time point were rarefied to 50000 and the second time point to 1000, while in version six, the total abundances at the first time point were rarefied to 1000 and the second time point to 50000. For negative binomial mode, versions three to six were further rarefied to either 5000 or 1000 (as per the lowest sequencing depth) at both time points. Microbes with BH-corrected model test q-values < 0.1 and BH-corrected post-hoc test q-values < 0.05 are regarded as significant. Negbin: negative binomial model; LM: linear model; rarefied: rarefied counts; TSS: total-sum scaling.

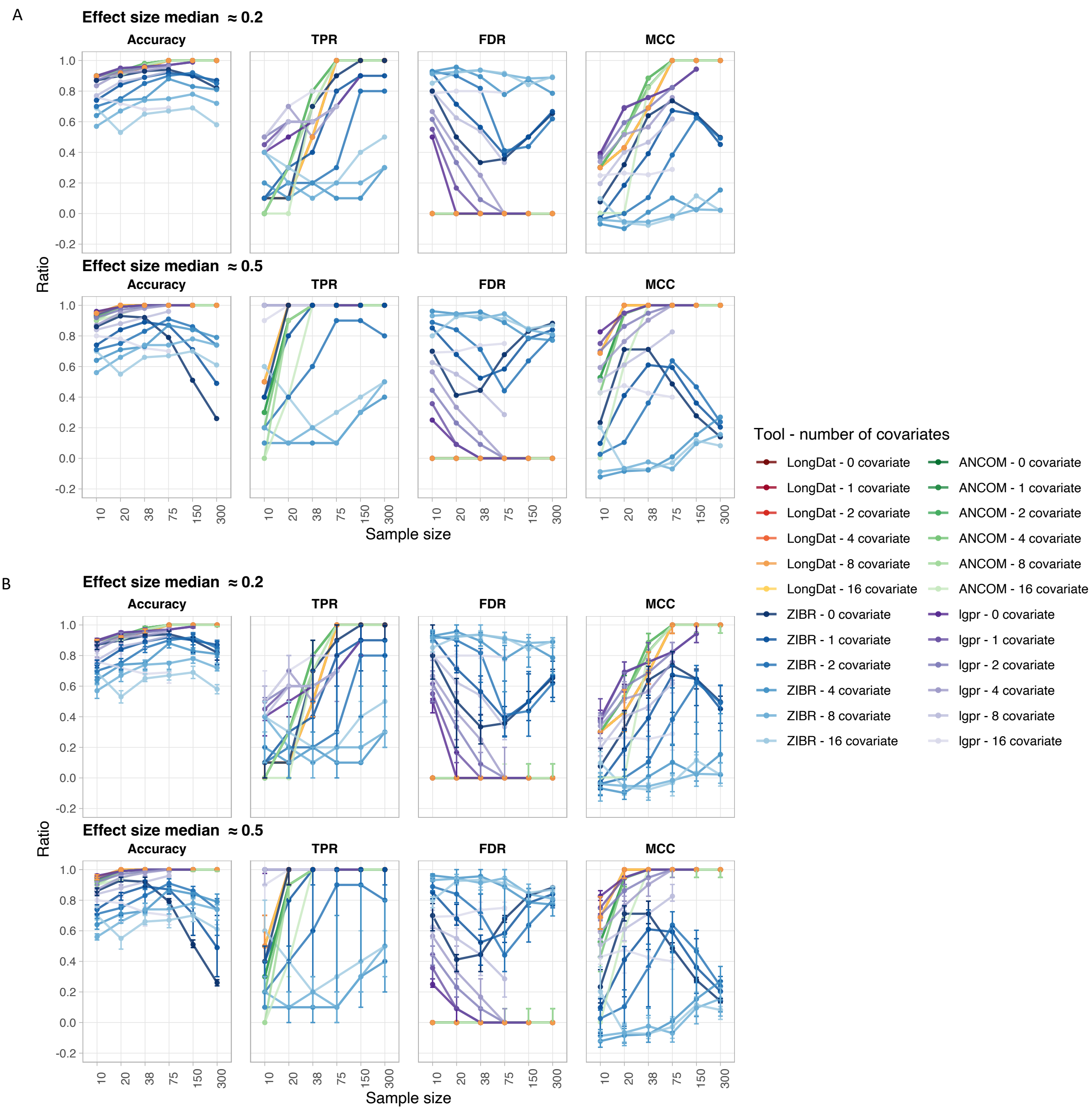

Fig. S10. Comparison of LongDat, ANCOM, ZIBR and lgpr on performance and power applied to microbiomeDASim-simulated longitudinal data with multiple covariates. The line plots show the medians of accuracy, true positive rate (TPR), false discovery rate (FDR), and Matthews correlation coefficient (MCC) without (A) and with (B) the first and third quartiles. The lower bars are the first quartiles, while the upper bars are the third quartiles. For each combination of sample size, effect size, covariate number, and tool, 25 simulations were performed.

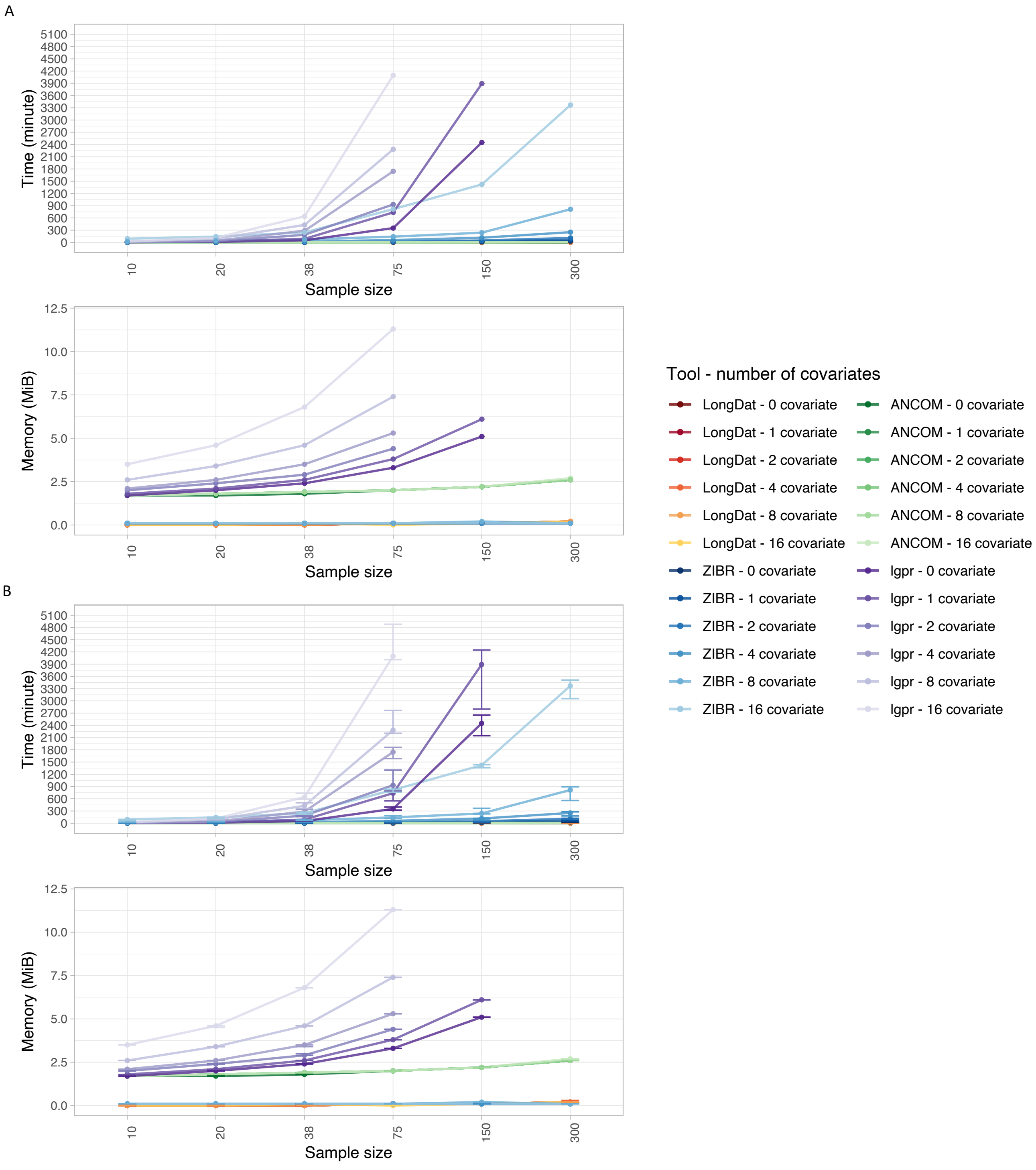

Fig. S11. Comparison of LongDat, ANCOM, ZIBR and lgpr on computational resource profiling applied to microbiomeDASim-simulated longitudinal data with multiple covariates. Line plots show the medians of runtime and total used memory required by LongDat and MaAsLin2 when run on simulated data with 100 features and various sample sizes without (A) and with (B) the first and third quartiles. The lower bars are the first quartiles, while the upper bars are the third quartiles. For each sample size, 50 simulations were performed. 1 mebibyte (MiB) ≈ 1.05 megabyte (MB).

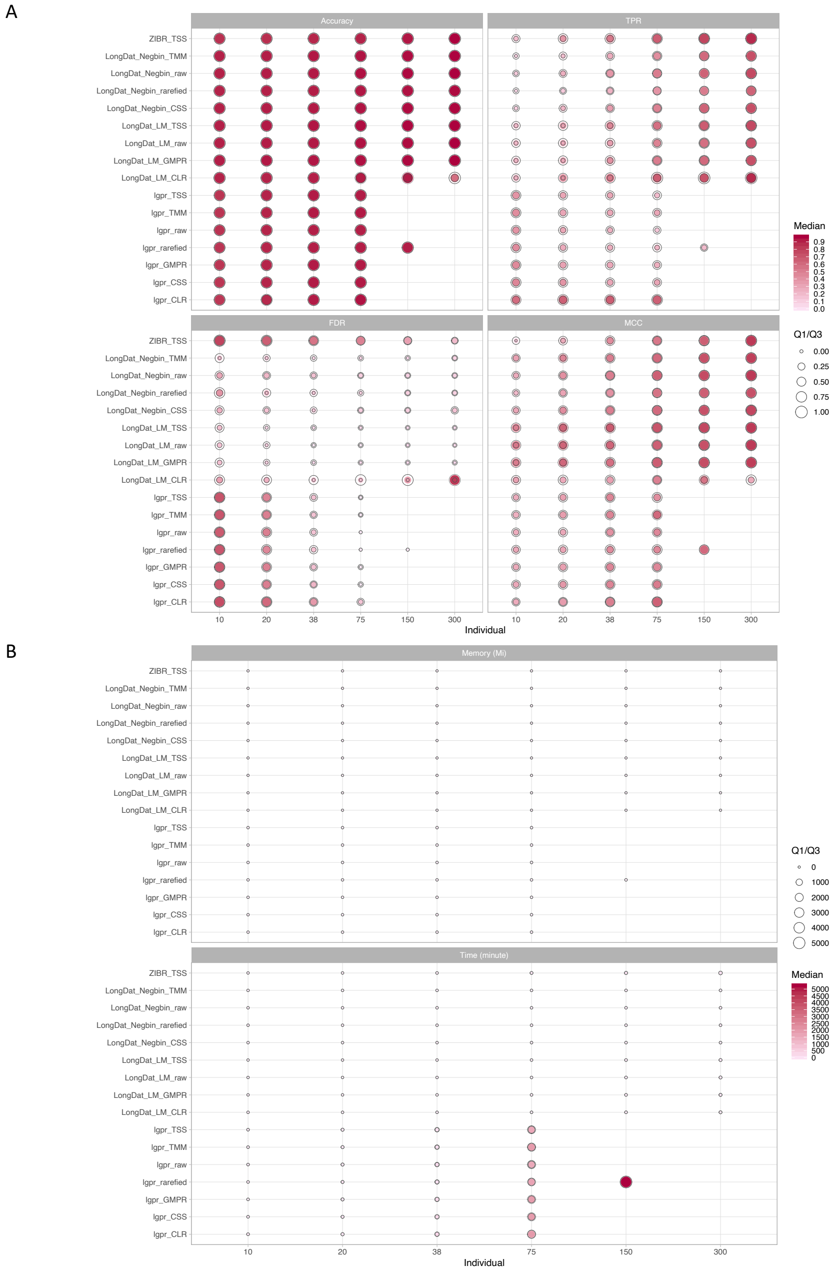

Fig. S12. Comparison of different modes of LongDat, lgpr, and ZIBR on performance and computational resource profiling applied to SparseDOSSA2-simulated longitudinal data with no covariate. The circles show the first quartiles, medians and third quartiles of (A) accuracy, true positive rate (TPR), false discovery rate (FDR), and Matthews correlation coefficient (MCC), and (B) runtime and total used memory. The filled circles (red) are the medians, while the inner and outer hollow circles are the first and third quartiles, respectively. Several blanks are left in the plot since lgpr couldn't finish the analyses with larger sample sizes within the time limit (96 hours) of the high-performance computing cluster we used. For each sample size, 200 simulations were performed (100 simulations of effect size median ≈ 0.2 Spearman's rho and 100 simulations of effect size median ≈ 0.5 Spearman's rho for time-varying features). 1 mebibyte (MiB) ≈ 1.05 megabyte (MB). Negbin: negative binomial model; LM: linear model; TMM: trimmed mean of M-values; raw: raw counts; rarefied: rarefied counts; CSS: cumulative-sum scaling; TSS: total-sum scaling; GMPR: geometric mean of pairwise ratios; CLR: centered log-ratio.

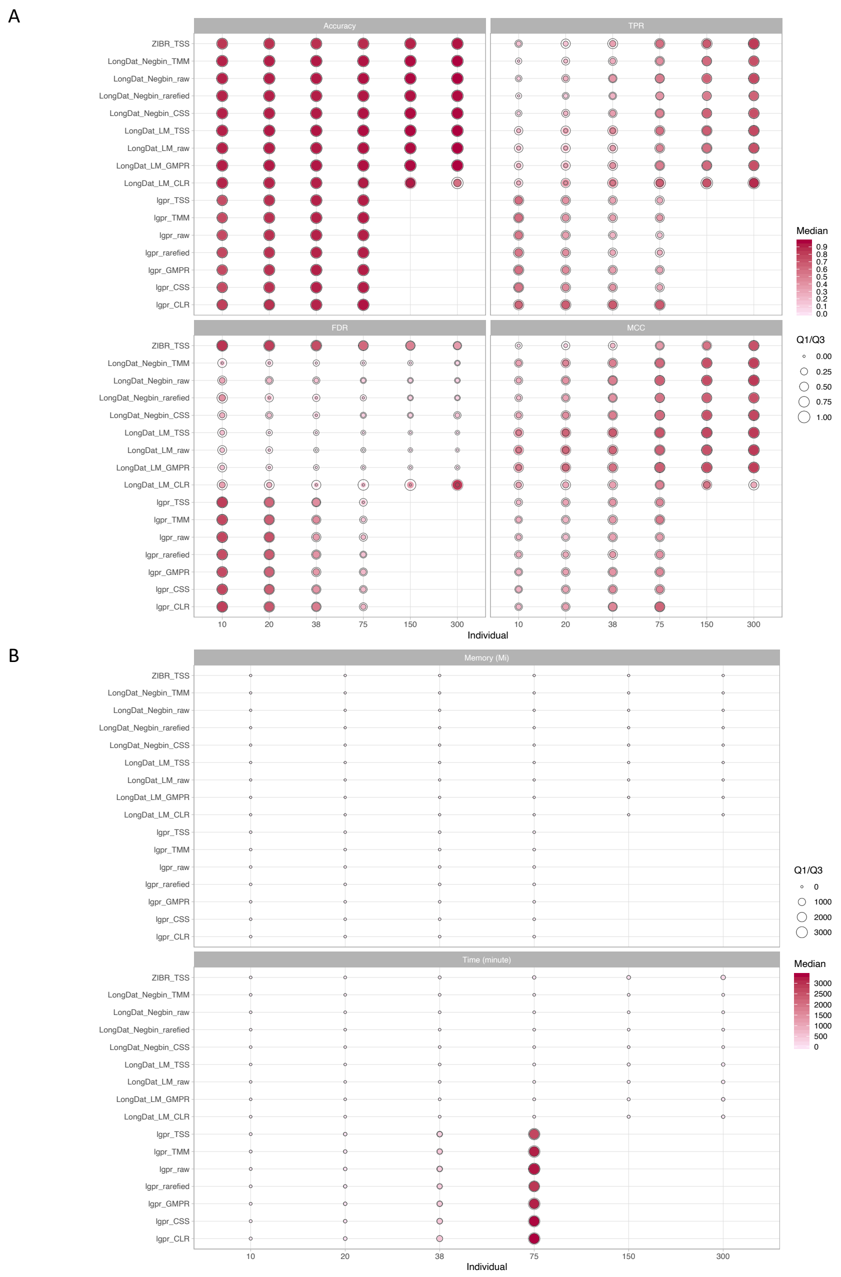

Fig. S13. Comparison of different modes of LongDat, lgpr, and ZIBR on performance and computational resource profiling applied to SparseDOSSA2-simulated longitudinal data with 1 covariate. The circles show the first quartiles, medians and third quartiles of (A) accuracy, true positive rate (TPR), false discovery rate (FDR), and Matthews correlation coefficient (MCC), and (B) runtime and total used memory. The filled circles (red) are the medians, while the inner and outer hollow circles are the first and third quartiles, respectively. Several blanks are left in the plot since lgpr couldn't finish the analyses with larger sample sizes within the time limit (96 hours) of the high-performance computing cluster we used. For each sample size, 200 simulations were performed (100 simulations of effect size median ≈ 0.2 Spearman's rho and 100 simulations of effect size median ≈ 0.5 Spearman's rho for time-varying features). 1 mebibyte (MiB) ≈ 1.05 megabyte (MB). Negbin: negative binomial model; LM: linear model; TMM: trimmed mean of M-values; raw: raw counts; rarefied: rarefied counts; CSS: cumulative-sum scaling; TSS: total-sum scaling; GMPR: geometric mean of pairwise ratios; CLR: centered log-ratio.

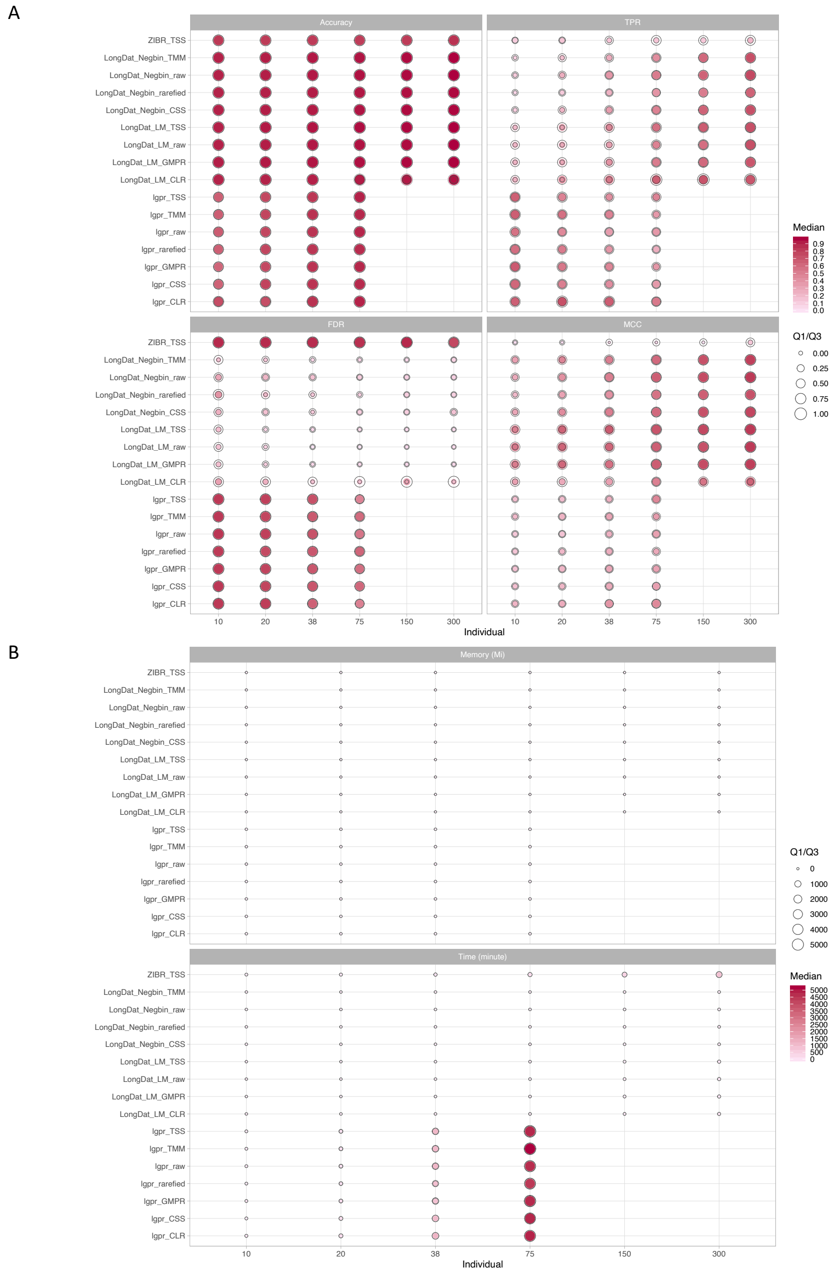

Fig. S14. Comparison of different modes of LongDat, lgpr, and ZIBR on performance and computational resource profiling applied to SparseDOSSA2-simulated longitudinal data with 4 covariates. The circles show the first quartiles, medians and third quartiles of (A) accuracy, true positive rate (TPR), false discovery rate (FDR), and Matthews correlation coefficient (MCC), and (B) runtime and total used memory. The filled circles (red) are the medians, while the inner and outer hollow circles are the first and third quartiles, respectively. Several blanks are left in the plot since lgpr couldn't finish the analyses with larger sample sizes within the time limit (96 hours) of the high-performance computing cluster we used. For each sample size, 200 simulations were performed (100 simulations of effect size median ≈ 0.2 Spearman's rho and 100 simulations of effect size median ≈ 0.5 Spearman's rho for time-varying features). 1 mebibyte (MiB) ≈ 1.05 megabyte (MB). Negbin: negative binomial model; LM: linear model; TMM: trimmed mean of M-values; raw: raw counts; rarefied: rarefied counts; CSS: cumulative-sum scaling; TSS: total-sum scaling; GMPR: geometric mean of pairwise ratios; CLR: centered log-ratio.

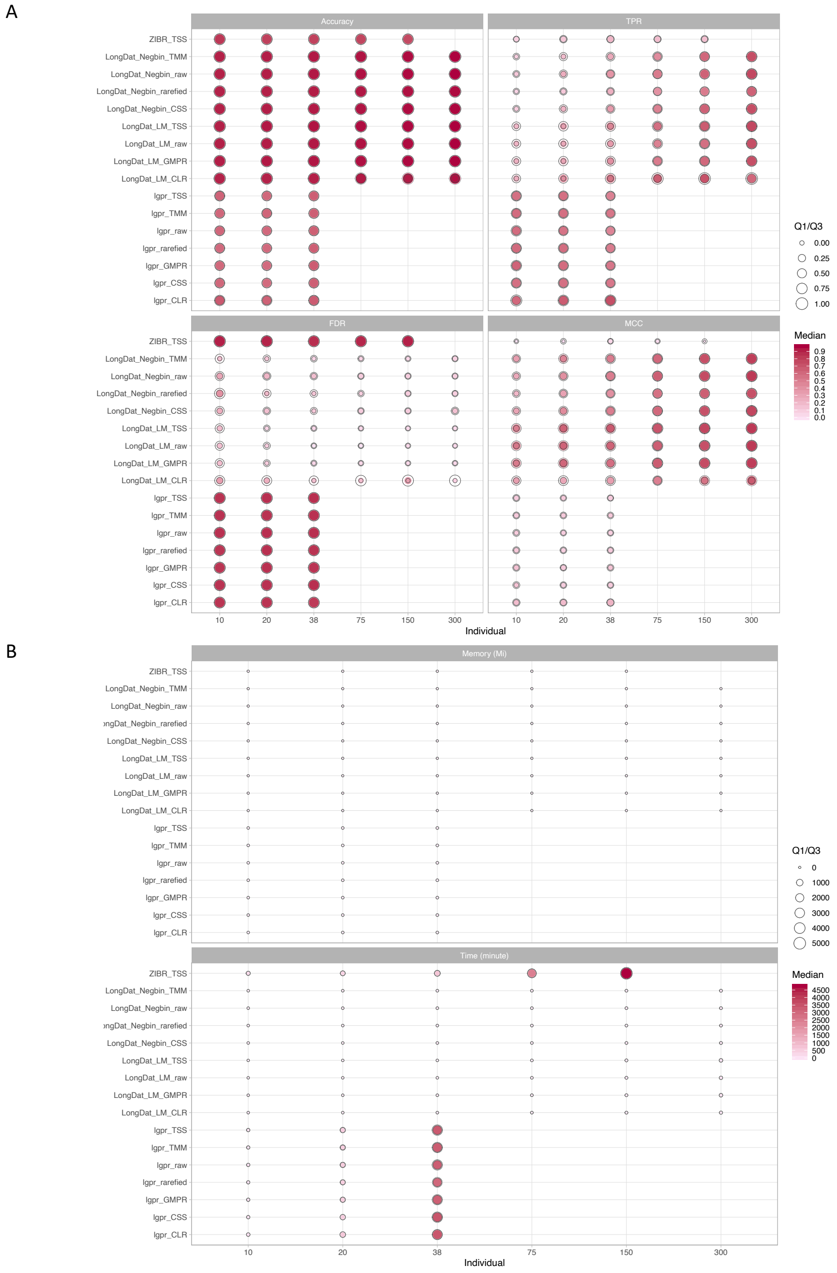

Fig. S15. Comparison of different modes of LongDat, lgpr, and ZIBR on performance and computational resource profiling applied to SparseDOSSA2-simulated longitudinal data with 16 covariates. The circles show the first quartiles, medians and third quartiles of (A) accuracy, true positive rate (TPR), false discovery rate (FDR), and Matthews correlation coefficient (MCC), and (B) runtime and total used memory. The filled circles (red) are the medians, while the inner and outer hollow circles are the first and third quartiles, respectively. Several blanks are left in the plot since lgpr and ZIBR couldn't finish the analyses with larger sample sizes within the time limit (96 hours) of the high-performance computing cluster we used. For each sample size, 200 simulations were performed (100 simulations of effect size median ≈ 0.2 Spearman's rho and 100 simulations of effect size median ≈ 0.5 Spearman's rho for time-varying features). 1 mebibyte (MiB) ≈ 1.05 megabyte (MB). Negbin: negative binomial model; LM: linear model; TMM: trimmed mean of M-values; raw: raw counts; rarefied: rarefied counts; CSS: cumulative-sum scaling; TSS: total-sum scaling; GMPR: geometric mean of pairwise ratios; CLR: centered log-ratio.

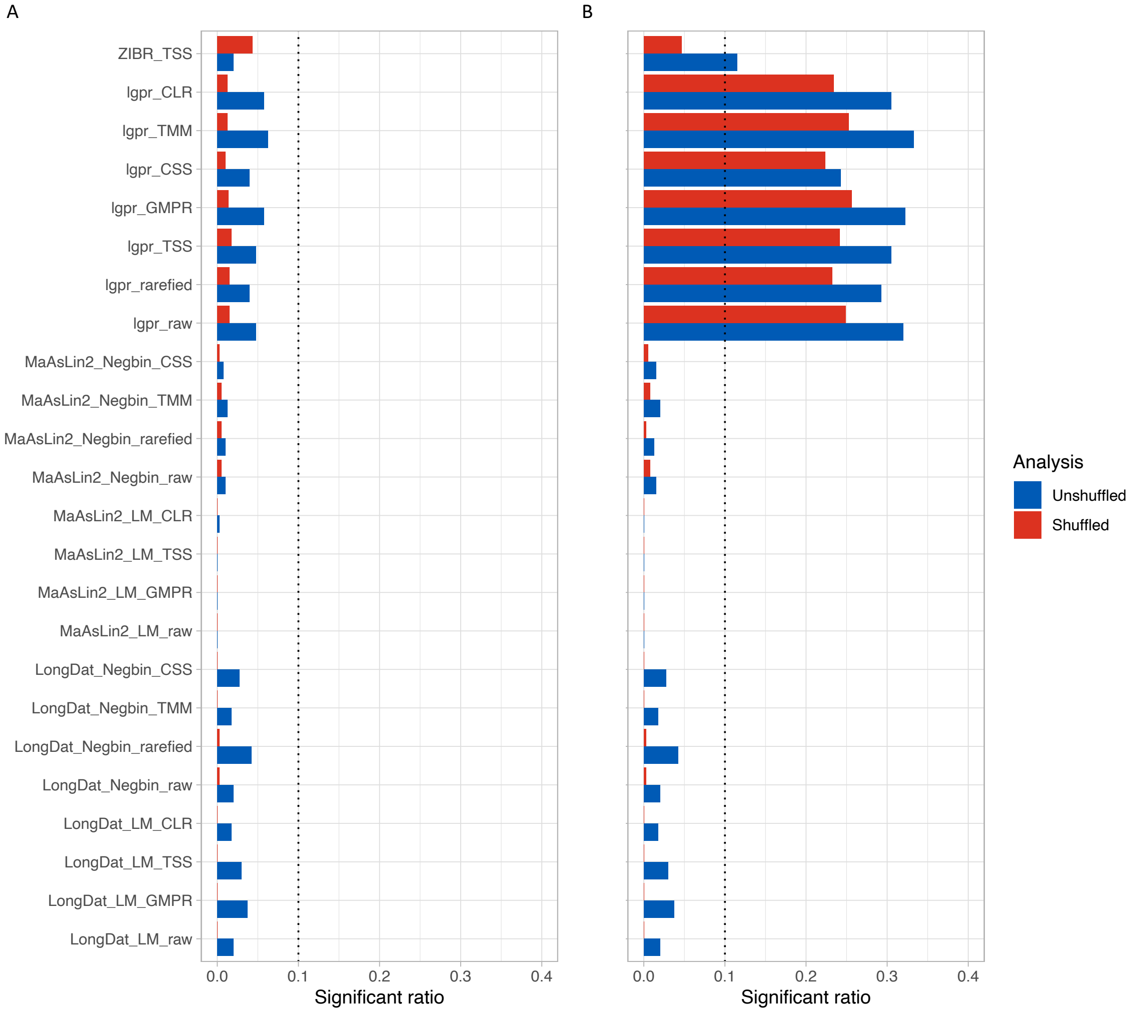

Fig. S16. Bar plots showing the ratio of significant association found in unshuffled and shuffled fasting microbiome data at genus level (Maifeld et al. 2021). This is testing the significance of the time variable in the original (unshuffled) and time-shuffled datasets, (A) without and (B) with covariates. The significant threshold (BH-corrected) is set at 0.1. For each shuffled test, 100 simulations were performed. Negbin: negative binomial model; LM: linear model; TMM: trimmed mean of M-values; raw: raw counts; rarefied: rarefied counts; CSS: cumulative-sum scaling; TSS: total-sum scaling; GMPR: geometric mean of pairwise ratios; CLR: centered log-ratio.

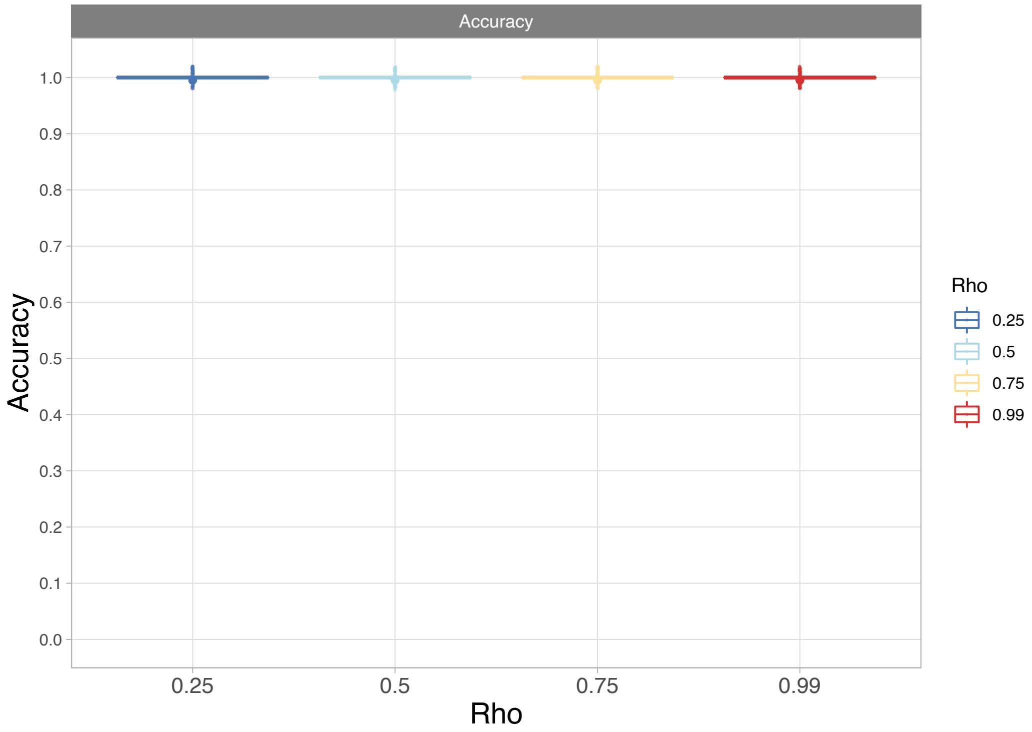

Fig. S17. Box plot showing the accuracy of LongDat applied to the negative-control data (simulated using microbiomeDASim) where time variable was shuffled. This is testing the significance of the shuffled time variable on the simulated features. Rho on the x-axis indicates the correlation between the dummy variable (see figure 4) and time. Note that in this figure, the correlation between the time variable and covariate was in fact destroyed because the time variable was shuffled. For each rho, 100 simulations were performed.

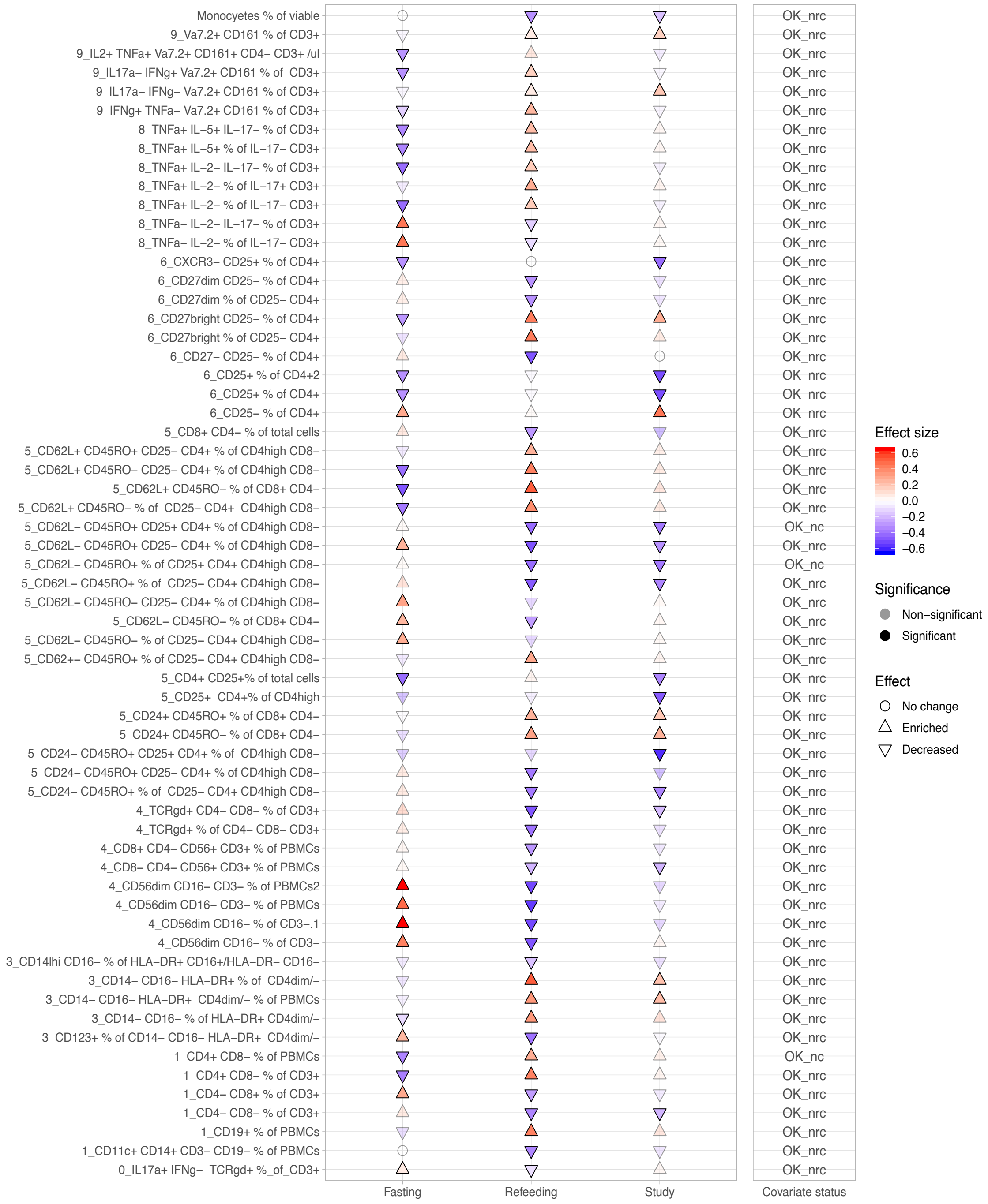

Fig. S18. The cuneiform plot on the left panel shows the immune cells (proportion data) that display significant differences in their abundance in at least one of the time intervals in the assessed study (Maifeld *et al.* 2021). "Fasting" indicates the time elapsed from day 0 to 7, while "refeeding" indicates day 7 to 90, and "study" indicates the overall duration from day 0 to 90. The right panel reports the covariate status of each feature as follows. *OK_nc*: OK and no covariate. Time/intervention has significant effect and there is no covariate. *OK_nrc*: OK and not reducible to any covariate. Time/intervention has significant effect, and its effect is independent of all tracked covariates. Features with BH-corrected model test q-values < 0.1 and BH-corrected post-hoc test q-values < 0.05 are regarded as significant.

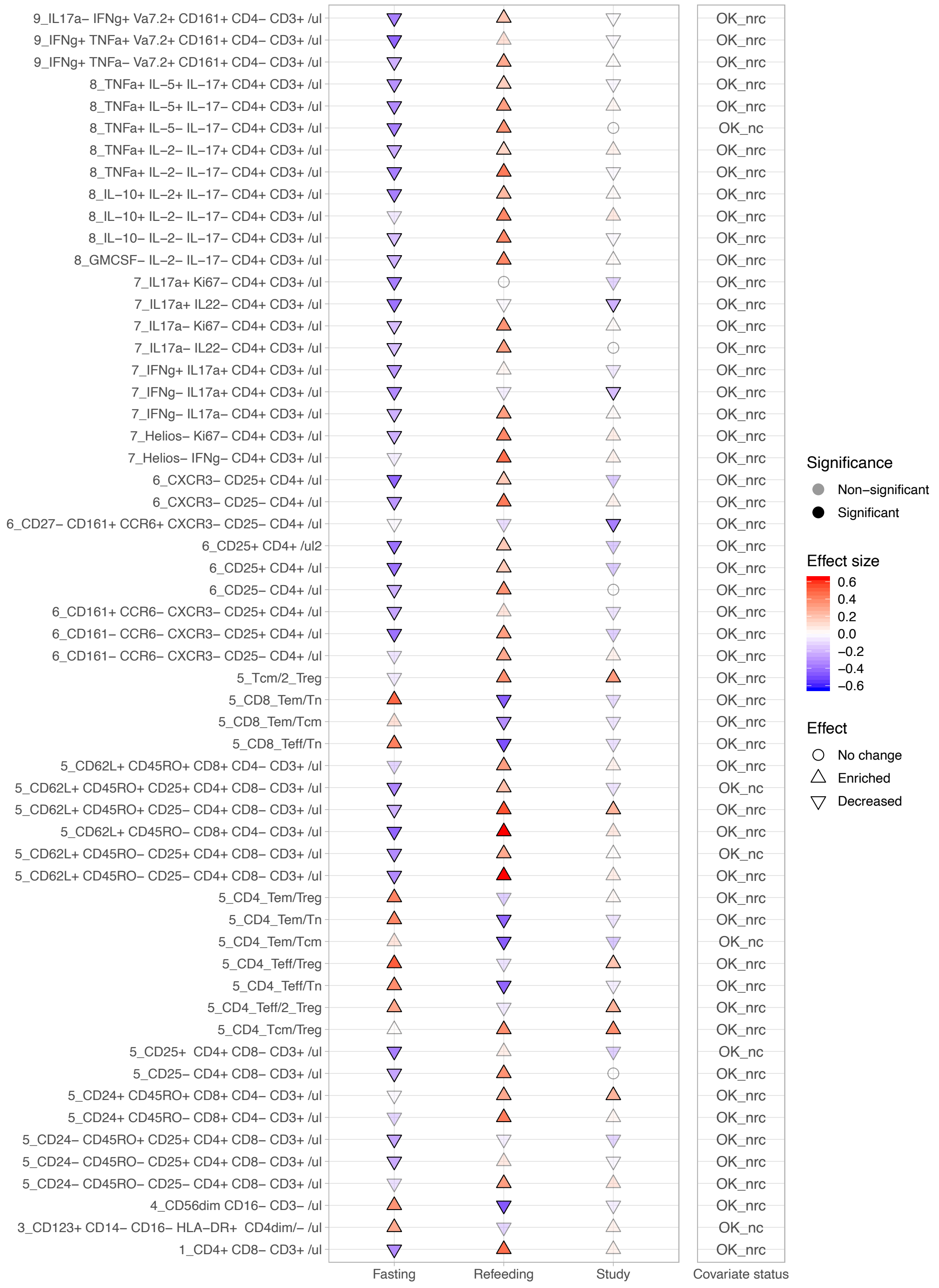

Fig. S19. The cuneiform plot on the left panel shows the immune cells (non-proportional data) that display significant differences in their abundance in at least one of the time intervals in the assessed study (Maifeld *et al.* 2021). "Fasting" indicates the time elapsed from day 0 to 7, while "refeeding" indicates day 7 to 90, and "study" indicates the overall duration from day 0 to 90. The right panel reports the covariate status of each feature as follows. *OK_nc*: OK and no covariate. Time/intervention has significant effect and there is no covariate. *OK_nrc*: OK and not reducible to any covariate. Time/intervention has significant effect, and its effect is independent of all tracked covariates. Features with BH-corrected model test q-values < 0.1 and BH-corrected post-hoc test q-values < 0.05 are regarded as significant.

References

Chen, E. Z., *et al.* (2016). "A two-part mixed-effects model for analyzing longitudinal microbiome compositional data." Bioinformatics **32**(17): 2611-2617.
Maifeld, A., *et al.* (2021). "Fasting alters the gut microbiome reducing blood pressure and body weight in metabolic syndrome patients." Nat Commun **12**(1): 1970.
Mallick, H., *et al.* (2021). "Multivariable association discovery in population-scale meta-omics studies." PLOS Computational Biology **17**(11): e1009442.
Mandal, S., *et al.* (2015). "Analysis of composition of microbiomes: a novel method for studying microbial composition." Microbial Ecology in Health and Disease **26**(1): 27663.
Timonen, J., *et al.* (2021). "lgpr: an interpretable non-parametric method for inferring covariate effects from longitudinal data." Bioinformatics **37**(13): 1860-1867.
